# Direct Position Determination of Unknown Signals in the Presence of Multipath Propagation

**DOI:** 10.3390/s18030892

**Published:** 2018-03-17

**Authors:** Jianping Du, Ding Wang, Wanting Yu, Hongyi Yu

**Affiliations:** National Digital Switching System Engineering and Technological Research Center, Zhengzhou 450002, China; dullenx@126.com (J.D.); wang_ding814@aliyun.com (D.W.); ywan1107@163.com (W.Y.)

**Keywords:** direct position determination, MUSIC algorithm, maximum likelihood estimation, convex QP, multi-path geolocation, active set algorithm

## Abstract

A novel geolocation architecture, termed “Multiple Transponders and Multiple Receivers for Multiple Emitters Positioning System (MTRE)” is proposed in this paper. Existing Direct Position Determination (DPD) methods take advantage of a rather simple channel assumption (line of sight channels with complex path attenuations) and a simplified MUltiple SIgnal Classification (MUSIC) algorithm cost function to avoid the high dimension searching. We point out that the simplified assumption and cost function reduce the positioning accuracy because of the singularity of the array manifold in a multi-path environment. We present a DPD model for unknown signals in the presence of Multi-path Propagation (MP-DPD) in this paper. MP-DPD adds non-negative real path attenuation constraints to avoid the mistake caused by the singularity of the array manifold. The Multi-path Propagation MUSIC (MP-MUSIC) method and the Active Set Algorithm (ASA) are designed to reduce the dimension of searching. A Multi-path Propagation Maximum Likelihood (MP-ML) method is proposed in addition to overcome the limitation of MP-MUSIC in the sense of a time-sensitive application. An iterative algorithm and an approach of initial value setting are given to make the MP-ML time consumption acceptable. Numerical results validate the performances improvement of MP-MUSIC and MP-ML. A closed form of the Cramér–Rao Lower Bound (CRLB) is derived as a benchmark to evaluate the performances of MP-MUSIC and MP-ML.

## 1. Introduction

We are interested in high precision positioning for shortwave signal sources in this paper. Two-step methods, such as the Angle Of Arrival (AOA) method, were usually used for shortwave signal positioning, and the methods provided a poor performance in a low Signal Noise Ratio (SNR) scenario. It has been shown that available prior knowledge on deterministic multi-path components can be beneficial for localization [1]. Kietlinski-Zaleski Jan presented techniques to benefit from signal reflections from known indoor features such as walls [2]. Inspired by those ideas, we propose a novel geolocation system architecture to locate the shortwave sources. This new architecture, termed “Multiple Transponders and Multiple Receivers for Multiple Emitters Positioning System (MTRE)”, uses multiple transponders and receivers with known locations to locate multiple narrow band signals. The raw signals are transferred “in band” (i.e., as a man-made multi-path) by the transponders, and there is no need for a network infrastructure or an out-of-band channel bandwidth, which are required in an up/down converter system. In order to avoid the interference between the receiving and the sending signals of a transponder, we use different polarization modes to isolate the signals. In an MTRE system, man-made multiple paths from an emitter to a receiver are made to improve the positioning precisions and extending the positioning range.

Multi-path propagation is a really important problem in outdoor and indoor positioning systems, and it is still the main source of estimation errors for range-based indoor localization approaches [3,4]. The recent research in dealing with multi-path either tries to detect these situations statistically based on the received signals [5,6] or to directly mitigate the corresponding errors with statistical techniques [7,8]. Some algorithms for indoor localization make use of, e.g., the cooperation of multiple agents to overcome multi-path situations [9]. Arrays were used for beam-forming to separate signals from different directions, and the multi-path positioning problem is simplified into a single path positioning problem [10,11]. Furthermore, location fingerprinting, e.g., Received Signal Strength (RSS)-based methods, has been widely used in harsh environments [12,13]. It makes use of a priori training signals in multiple regions of the environment to train a classification algorithm [14]. However, the required training phase, as well as the missing flexibility w.r.t. changes in the environment may limit its application.

Most of the above literature focused on the UWB signals and indoor positioning applications, and two-step approaches were adopted to locate the emitters. The very high bandwidth of the UWB signal translates into very good time resolution and makes the UWB signal resistant to multi-path. It is possible to extract parameters, e.g., RSS, Time Of Arrival (TOA), AOA, Time Difference Of Arrival (TDOA) and Frequency Difference Of Arrival (FDOA), from UWB signals in the presence of multi-path propagation and to locate the emitter based on those parameters [15,16]. However, narrow-band systems have a low time resolution, and it is difficult to get the measurements in the first step.

The Direct Position Determination (DPD) methods were proposed in [17] for single narrow band signal positioning and in [18] for multiple narrow band signal positioning. A DPD approach collects data at all sensors together and uses both the array responses and the Times Of Arrival (TOA) at each array, in contradiction with the two separate steps: parameter measuring and location determination. From the optimization theory point of view, two-step methods are sub-optimal, since the parameter estimation in the first phase is done independently, without considering the constraints that the measurements must correspond to the same source position. DPD methods overcame the problem of associating estimated parameters with their relevant sources and was shown to outperform two-step methods, especially in low SNR scenarios [19].

There have been only a few attempts to improve the accuracy of emitter positioning in the presence of multi-path propagation under the DPD framework. Most of the existing DPD methods were developed for a single-path channel in which the multi-path was modeled as additive noise [20]. In [20], the single path DPD was tested with a channel with two paths scenario and showed improved performance over two-step methods. The DPD with small local scattering was studied in [21,22]. In a scattering scenario, sensors were affected by a set of virtual emitters, which were placed randomly in close proximity to the real emitter. It was assumed that the positions of the virtual emitters were i.i.d, and each position forms a 3D Gaussian distribution. Odel Bialer and Dan Paphaeli and Anthony J. Weiss [23,24] proposed a positioning algorithm for a dense multipath environment. Each received signal was obtained by convolving the transmitted pulse with a channel impulse response, and only the first arrival cluster (the direct path) was taken into consideration in their work. The signals reflected from other objects were not modeled in their work.

Papakonstantinou and Slock [25,26] considered a simplified single-bounce multipath model. The model assumed that the transmitted signal did not bounce over more than one scatter. They jointly estimated the position of the target and scatters. They studied the single emitter positioning problem in the presence of multi-path propagation and assumed that the waveform of signal and path attenuations were known in advance. Miljko and Vucic [27] proposed a novel direct geolocation of an Ultra WideBand (UWB) source in the presence of multi-path using the MUltiple SIgnal Classification (MUSIC) method and focusing matrices. Only one emitter was taken into consideration, and the path attenuations are known in advance in their work. Bar-Shalom et al. [28,29] proposed a transponder-added Single Platform Geolocation (SPG) model. A single emitter and single receiver were assumed in the SPG model. They stated that the SPG model achieved a similar performance to the multiple-RX DPD algorithm. The multiple-RX DPD algorithm mentioned in their works assumed that the transponders were replaced by the receivers directly. In a weak signal location application, a single receiver cannot receive signals from all transponders stably. Some paths may be blocked or disrupted. Multiple emitters, multiple transponders and multiple receivers need to be taken into consideration in a weak signal positioning application.

All unknown parameters should be estimated together in a DPD model, and this leads to a large-scale parameter searching. MUSIC methods calculate the spatial spectrum of each candidate position rather than the combinations of all emitter positions. Amar et al. [30] studied that multiple known and unknown radio-frequency signals under the LoS (Line of Sight) channel assumption. A simplified MUSIC algorithm was adopted to avoid the large-scale parameter searching. The cost function in [30] maximized the projection of the array manifold onto the signal subspace rather than minimizing the projection onto the noise subspace. The simplified cost function took advantage of the maximization of the convex Quadratic Programming (QP) with linear constraints, and the eigenvalue structure was adopted to avoid the searching of path attenuation parameters in their work. The simplified MUSIC worked well in an LoS propagation context, but it had a poor performance in a multi-path propagation scenario due to the singularity of the array manifold. Minimizing the projection of the array manifold onto the noise subspace overcomes the shortcomings of a signal subspace projection method. However, the eigenvalue system fails to resolve the minimization programming.

Existing DPD methods mainly focus on narrow-band signal positioning [18,29,31,32,33] and usually assume that the carrier phase does not carry the propagation delay information. Complex channel attenuations were estimated to eliminate the influence of carrier phase misalignment in a narrow-band signal positioning method. We point out that the narrow-band assumption will lose the phase information in an LoS positioning application, and it will not be able to locate emitters at all in a multi-path positioning application. We add constraints that path attenuations are nonnegative real numbers in our model. In an existing DPD model, path attenuations are complex numbers and have only one equation constraint (the norm of path attenuations is one), and the Lagrange-multiplier method is very effective at solving the optimization with equation constraints [34]. However, it is difficult to solve an optimization with inequality constraints (the path attenuations should be greater than zero). We are required to design an efficient algorithm to solve the QP with inequality constraints.

The performance of a MUSIC method is determined by the precision of the covariance matrix estimation. In a time-sensitive application, the number of snapshots is not enough, and it is difficult to estimate the covariance matrix precisely. The maximum likelihood method maximizes the likelihood function of the received data rather than estimating the covariance matrix, and it achieves a better performance than that of the MUSIC method. However, the dimension of the searching space turns out to be unacceptable in the maximum likelihood method.

Our motivation is to develop a simple and accurate positioning model and corresponding algorithms for the case of unknown waveform signals and multi-path environment. We establish a Multi-path Propagation (MP)-DPD model for the scenario of multiple emitters, multiple transponders and multiple receiving arrays. It can be viewed as a modified and extended version of the SPG model proposed in [29]. The MP-DPD reduces the risk of paths being blocked or disrupted and fixes the constraints on path attenuations. Multiple emitters can be simultaneously positioned in the MP-DPD model, as well. MP-MUSIC and MP-ML methods are proposed to reduce the time consumption of the optimization. The numerical results and the Cramér–Rao Lower Bound (CRLB) analysis show that the MP-MUSIC method has a lower computing complexity than MP-ML, especially in the case of a complex multipath scenario. The MP-ML method is more precise than MP-MUSIC, especially in the case of positioning with limited snapshots. An Active Set Algorithm (ASA) for the MP-MUSIC and an iterative algorithm for the MP-ML are developed to reduce the computational complexity of the methods further. Numerical results demonstrate that the MP-MUSIC and MP-ML proposed in this paper outperform the conventional methods.

The paper is organized as follows: Section 2 outlines the problem formulation, and an MP-DPD model is established in this section. The MP-MUSIC method, the MP-ML method and corresponding algorithms are proposed in Section 3 and Section 4, Numerical performance examples of these algorithms are given in Section 5. The final conclusions are given in Section 6. Finally, the detailed descriptions of the ASA algorithm, the iterative algorithm for MP-ML method and the derivation of the CRLB are provided in the Appendix.

## 2. Problem Formulation

**Figure 1:**
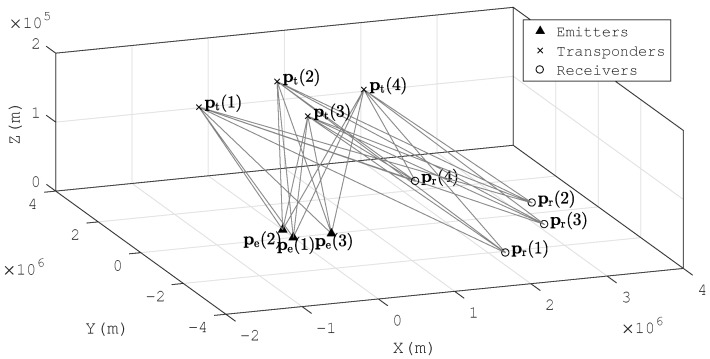
Multiple-path positioning problem with static transponders/receivers.

Consider that there are *D* emitters located at $p_{e}={\left[p_{e}^{T}\left(1\right),p_{e}^{T}\left(2\right),\ldots ,p_{e}^{T}\left(D\right)\right]}^{T}$ and *L* passive transponders placed at $p_{t}={\left[p_{t}^{T}\left(1\right),p_{t}^{T}\left(2\right),\ldots ,p_{t}^{T}\left(L\right)\right]}^{T}$. The signals transmitted by the emitters are reflected by the transponders and intercepted by *N* receiving arrays. Each array includes *M* antennas. The centers of the arrays are located at $p_{r}={\left[p_{r}^{T}\left(1\right),p_{r}^{T}\left(2\right),\ldots ,p_{r}^{T}\left(N\right)\right]}^{T}$. It is assumed that the locations of the transponders and the receiving arrays are known a priori and that the signal waveforms are unknown. The scenario is depicted in Figure 1.

Denote the signal propagation delay between the *d*-th emitter and the *ℓ*-th transponder by ${\bar{\tau }}_{d\ell }$. Denote:

(1) $${\tilde{\tau }}_{\ell n}={\left[{\tilde{\tau }}_{\ell n1},{\tilde{\tau }}_{\ell n1},\ldots ,{\tilde{\tau }}_{\ell nM}\right]}^{T},$$

where ${\tilde{\tau }}_{\ell nm}$ is the propagation delay between the *ℓ*-th transponder and the *m*-th antenna in the *n*-th receiving array. ${\tilde{\tau }}_{\ell n}$ is an M×1 column vector, which represents the propagation delays from the *ℓ*-th transponder to the *n*-th receiving array. ${\tilde{\tau }}_{\ell n}$ is known in advance, and it is independent of the emitter positions.

The path attenuation from the *d*-th emitter to the *n*-th receiving array, which is reflected by the *ℓ*-th transponder, is denoted by $\alpha_{d\ell n}$. The path attenuation coefficients are assumed as non-negative real numbers, and the rationality of the assumption will be discussed in detail in Section 3.1.2. We assume that the antennas in a receiving array are uniform, and all antennas in an array share the same path attenuation coefficient.

The time-domain model of the signals that are received by the *n*-th receiving array is:

(2) $${\bar{r}}_{n}\left(t\right)=\sum_{\ell =1}^{L}\sum_{d=1}^{D}\left[\alpha_{d\ell n}{\bar{s}}_{d}\left(t-{\tilde{\tau }}_{\ell n}-{\bar{\tau }}_{d\ell }-t_{d}\right)\right]+\bar{n}\left(t\right),$$

where ${\bar{r}}_{n}\left(t\right)$ is an M×1 column vector, which represents *M* snapshots at time *t* of the *n*-th receiving array. ${\bar{s}}_{d}\left(t\right)$ is an M×1 column vector, which represents *M* snapshots of the *d*-th source signal at time vector $t≜t-{\tilde{\tau }}_{\ell n}-{\bar{\tau }}_{d\ell }-t_{d}$. $\bar{n}\left(t\right)$ is an M×1 noise vector at time *t*. 0≤t≤T, and $t_{d}$ is the unknown transmit time of the emitter *d*. We assume that the path attenuation, $\alpha_{d\ell n}$, remains constant during the observation time interval. This paper mainly focuses on the positioning of deterministic, but unknown signals. It is assumed that source signals are independent of one another, and there is no further requirement for the code or waveform of the signals. The frequency-domain model for the *k*-th DFT coefficients is given by:

(3) $$r_{n}\left(k\right)=\sum_{\ell =1}^{L}\sum_{d=1}^{D}\alpha_{d\ell n}{\tilde{a}}_{\ell n}\left(k\right)e^{-i\omega_{k}{\bar{\tau }}_{d\ell }}s_{d}\left(k\right)+n\left(k\right),$$

where:

(4) $$\begin{array}{cc} {\tilde{a}}_{\ell n}\left(k\right) & =e^{-i\omega_{k}{\tilde{\tau }}_{\ell n}},\\ {\overset{ˇ}{s}}_{d}\left(k\right) & =s_{d}\left(k\right)e^{-i\omega_{k}t_{d}},\\ \omega_{k} & =\frac{2\pi k}{T},\\ k & =1,2,\cdots ,K, \end{array}$$

where $s_{d}\left(k\right)$ is the *k*-th Fourier coefficient of the *d*-th source signal ${\bar{s}}_{d}\left(t\right),t\in \left[0,T\right]$. $r_{n}\left(k\right)$ and n(k) are M×1 vectors of the *k*-th Fourier coefficients of ${\bar{r}}_{n}\left(t\right)$ and $\bar{n}\left(t\right)$. ${\tilde{a}}_{\ell n}\left(k\right)$ is an M×1 vector, which denotes the generalized array response of the *n*-th receiver at frequency $\omega_{k}$. Make (3) into matrix form:

(5) $$r\left(k\right)=A\left(k\right)\overset{ˇ}{s}\left(k\right)+n\left(k\right),$$

where:

$$\begin{array}{cc} r(k) & ≜{\left[r_{1}^{T}\left(k\right),r_{2}^{T}\left(k\right),\ldots ,r_{N}^{T}\left(k\right)\right]}^{T},\\ r_{n}^{T}\left(k\right) & ={\left[r_{n1}^{T}\left(k\right),r_{n2}^{T}\left(k\right),\ldots ,r_{nM}^{T}\left(k\right)\right]}^{T},\\ A(k) & ≜\tilde{A}\left(k\right)V\left(k\right)\alpha ,\\ \tilde{A}\left(k\right) & =\left[\begin{array}{cccc} {\tilde{A}}_{1}\left(k\right) & 0 & \cdots & 0\\ 0 & {\tilde{A}}_{2}\left(k\right) & \cdots & 0\\ \vdots & \vdots & \ddots & \vdots \\ 0 & 0 & \cdots & {\tilde{A}}_{N}\left(k\right) \end{array}\right],\\ {\tilde{A}}_{n}\left(k\right) & =[{\tilde{a}}_{1n}\left(k\right),{\tilde{a}}_{2n}\left(k\right),\ldots ,{\tilde{a}}_{Ln}\left(k\right)], \end{array}$$

$$\begin{array}{cc} V(k) & =I_{N}⊗\bar{V}\left(k\right),\\ \bar{V}\left(k\right) & =[{\bar{V}}_{1}\left(k\right),{\bar{V}}_{2}\left(k\right),\ldots ,{\bar{V}}_{D}\left(k\right)],\\ {\bar{V}}_{d}\left(k\right) & =\mathrm{diag}(\left[e^{-i\omega_{k}{\bar{\tau }}_{d1}},e^{-i\omega_{k}{\bar{\tau }}_{d2}},\ldots ,e^{-i\omega_{k}{\bar{\tau }}_{dL}}\right]),\\ \alpha & =\left[\begin{array}{c} \alpha_{1}\\ \alpha_{2}\\ \vdots \\ \alpha_{N} \end{array}\right],\\ \alpha_{n} & =\left[\begin{array}{cccc} \alpha_{1n} & 0 & \cdots & 0\\ 0 & \alpha_{2n} & \cdots & 0\\ \vdots & \vdots & \ddots & \vdots \\ 0 & 0 & \cdots & \alpha_{Dn} \end{array}\right],\\ \alpha_{dn} & ={\left[\alpha_{d1n},\alpha_{d2n},\ldots ,\alpha_{dLn}\right]}^{T},\\ \overset{ˇ}{s}\left(k\right) & ≜{\left[{\overset{ˇ}{s}}_{1}\left(k\right),{\overset{ˇ}{s}}_{2}\left(k\right),\ldots ,{\overset{ˇ}{s}}_{D}\left(k\right)\right]}^{T}. \end{array}$$

where ⊗ is the Kronecker product and $I_{N}$ is an identify matrix with a size of N×N.

Denote the second moments of variables by:

(6) $$\begin{array}{cc} \;E\{n\left(k\right)n^{H}\left(k\right)\} & =\sigma^{2}I_{MN}≜\Sigma ,\\ E\{n\left(k\right)n{\left(k\right)}^{T}\} & =0, \end{array}$$

(7) $$\begin{array}{cc} R(k) & ≜E\left\{r\left(k\right)r^{H}\left(k\right)\right\}=A\left(k\right)\Lambda \left(k\right)A^{H}\left(k\right)+\Sigma , \end{array}$$

(8) $$\begin{array}{cc} \Lambda (k) & ≜E\{\overset{ˇ}{s}\left(k\right){\overset{ˇ}{s}}^{H}\left(k\right)\}, \end{array}$$

where $I_{MN}$ is an identity matrix with a size of MN×MN. R(k) is a covariance matrix of received signals at frequency $\omega_{k}$. σ is the noise standard deviation. The observed signal of each antenna $\bar{r}\left(t\right)$ is partitioned into *J* sections, and each section is Fourier transformed. The *k*-th Fourier coefficient of the *j*-th section is denoted by $r_{j}\left(k\right)$. The covariance matrix at frequency $\omega_{k}$ is estimated by:

(9) $$\begin{array}{cc} \hat{R}\left(k\right) & =\frac{1}{J}\sum_{j=1}^{J}r_{j}\left(k\right)r_{j}^{H}\left(k\right). \end{array}$$

The relationship between the received signals and emitter positions has been established in (5), and it is named the MP-DPD model. The MP-DPD model optimizes the emitter positions directly to achieve a more accurate estimation. Two DPD methods are proposed under the MP-DPD framework:

- MP-MUSIC method;
- MP-ML method.

The array manifold projection onto the noise subspace is adopted as the cost function in the MP-MUSIC method, and the likelihood function of the received signals is adopted in the MP-ML method. If the number of snapshots is sufficient, MP-MUSIC consumes less time than MP-ML without degrading the performance. Besides, if the number of snapshots is not enough, MP-ML obtains more precise position estimations than MP-MUSIC.

## 3. MP-MUSIC Method

We analyze the shortcomings of the existing MUSIC method in the multi-path propagation positioning firstly and establish an MP-MUSIC model that is suitable for the multi-path environment positioning. Finally, the corresponding algorithm is given at the end of this section.

### 3.1. The Limitation of Existing MUSIC Methods

We introduce the Signal Subspace Projection MUSIC (SSP-MUSIC) method, which was commonly used in the DPD model firstly, and develop a Noise Subspace Projection MUSIC (NSP-MUSIC) method to overcome the shortage of SSP-MUSIC. Finally, we discuss the performances of SSP-MUSIC and NSP-MUSIC, which were adopted in a multi-path positioning application.

#### 3.1.1. SSP-MUSIC

Alan Amar and Anthony J. Weiss studied the positioning problem of multiple unknown radio-frequency signals in [30]. The MUSIC method for the LoS propagation positioning was proposed in their works. Alan Amar maximized the manifold projection onto the signal subspace rather than minimizing the projection onto the noise subspace. The programming model of Amar’s method was defined as:

(10) $$\left[{\hat{p}}_{e},\hat{\alpha }\right]=\arg\max F\left(p_{e},\alpha \right)=\alpha^{H}D\left(p_{e}\right)\alpha ,$$

(11) $$s.t.\left\{\begin{array}{c} {∥\alpha ∥}_{F}^{2}=1,\\ \alpha \in C^{ND}, \end{array}\right.$$

where:

(12) $$\begin{array}{cc} D(p_{e}) & ≜H^{H}\left[\sum_{k=1}^{K}\Gamma^{H}\left(k\right)U_{s}\left(k\right)U_{s}^{H}\left(k\right)\Gamma \left(k\right)\right]H, \end{array}$$

(13) $$\begin{array}{cc} H & ≜I_{N}⊗1_{M}, \end{array}$$

where α is the path attenuation vector, $C^{ND}$ is the set of complex column vectors with the length of ND, ${∥\cdot ∥}_{F}$ is the Frobenius norm of a matrix, $p_{e}$ is the vector of emitter positions, $I_{N}$ stands for the N×N identity matrix, $1_{M}$ stands for an M×1 column vector of ones, *M* stands for the number of antennas of each array, Γ(k) is the array manifold matrix at frequency $\omega_{k}$, *N* is the number of receivers, *D* is the number of emitters, *K* is the frequency points of received signals and $U_{s}\left(k\right)$ is made up of the eigenvectors of the covariance matrix of received signals corresponding to the *D* largest eigenvalues. The other parameter notations can be found in [30].

Since (10) is a quadratic convex optimization with linear constraints in the complex field, the maximum of the cost function is the maximal eigenvalue of the matrix $D(p_{e})$[31]; thus, the optimal cost function value is $F^{*}\left(p_{e}\right)=\lambda_{\max}\left\{D\left(p_{e}\right)\right\}$, where $\lambda_{\max}\left\{\cdot \right\}$ represents the maximal eigenvalue of a matrix. Benefiting from the simplified cost function and the eigenvalue system, Amar’s method reduced the searching dimension from 2DK+2(N−1)D+3 to three.

#### 3.1.2. NSP-MUSIC

A noise subspace projection MUSIC method is proposed in this paper to remove the simplification of the SSP-MUSIC. NSP-MUSIC minimizes the manifold projection onto the noise subspace:

(14) $$\left[\hat{p_{e}},\hat{\alpha }\right]=\arg\min\bar{F}\left(p_{e},\alpha \right)=\alpha^{H}H^{H}\sum_{k=1}^{K}\left\{\Gamma^{H}\left(k\right)\left[I-U_{s}\left(k\right)U_{s}^{H}\left(k\right)\right]\Gamma \left(k\right)\right\}H\alpha .$$

Reorganize the items in (14):

(15) $$\left[{\hat{p}}_{e},\hat{\alpha }\right]=\arg\max\tilde{F}\left(p_{e},\alpha \right)=\frac{1}{\alpha^{H}I\left(p_{e}\right)\alpha -\alpha^{H}D\left(p_{e}\right)\alpha },$$

where:

(16) $$I\left(p_{e}\right)≜H^{H}\left[\sum_{k}^{K}\Gamma^{H}\left(k\right)\Gamma \left(k\right)\right]H,$$

and $D(p_{e})$ has been defined in (12). SSP-MUSIC is viewed as a simplified version of (15). In a direction finding application, it is assumed that the path attenuation of each antenna in an array has been normalized in advance, and $\alpha^{H}I\left(p\right)\alpha$ is a known constant item. The dropping of the constant item in an optimization is reasonable. However, in a multi-path positioning application, $\alpha^{H}I\left(p\right)\alpha$ changes with the change of α, and the dropping of $\alpha^{H}I\left(p\right)\alpha$ is unreasonable.

Following the standard noise subspace MUSIC method and the model for the multi-path positioning, we define the cost function of NSP-MUSIC:

(17) $$\left[{\hat{p}}_{e},\hat{\alpha }\right]=\arg\max Q\left(p_{e},\alpha \right)=\frac{1}{\sum_{k=1}^{K}a^{H}\left(k\right)\left[I_{MN}-U_{s}\left(k\right)U_{s}^{H}\left(k\right)\right]a\left(k\right)},$$

where $I_{MN}$ is an identity matrix with a size of MN×MN. $U_{s}\left(k\right)$ is a matrix consisting of the eigenvectors of $R_{k}$ corresponding to the *D* largest eigenvalues. $p_{e}$ and α are decision making variable vectors representing the candidate emitter positions and the corresponding path attenuations. In order to facilitate a unique solution, we assume that the norm of α is one. a(k) is the array manifold vector for $p_{e}$ at frequency $\omega_{k}$. Unfortunately, the cost function requires an LN−1+3 dimensional searching, and it is difficult to get the optimal solution over such a high dimensional space.

Note that the *d*-th column of matrix A(k) in (5) is denoted by:

(18) $$a_{d}\left(k\right)=\tilde{A}\left(k\right)\left[I_{N}⊗{\bar{V}}_{d}\left(k\right)\right]\alpha_{d}≜\Gamma_{d}\left(k\right)\alpha_{d},$$

where $\alpha_{d}≜{\left[\alpha_{d1}^{T},\alpha_{d2}^{T},\ldots ,\alpha_{dN}^{T}\right]}^{T}$ represent attenuations of paths from the *d*-th emitter. The vector a(k) of a candidate emitter in the MUSIC algorithm (17) is similar to $a_{d}\left(k\right)$, but the position of the *d*-th emitter is replaced by the candidate emitter position $p_{e}$. Denote $\Gamma \left(k\right)≜\Gamma_{d}\left(k\right)$ to simplify the explanation, and substitute (18) into (17):

(19) $$\left[\hat{p_{e}},\hat{b}\right]=\arg\max Q\left(p_{e},\alpha \right)=\frac{1}{\alpha^{H}E\left(p_{e}\right)\alpha },$$

(20) $$s.t.\left\{\begin{array}{c} E\left(p_{e}\right)=\sum_{k=1}^{K}\Gamma^{H}\left(k\right)\left[I_{MN}-U_{s}\left(k\right)U_{s}^{H}\left(k\right)\right]\Gamma \left(k\right),\\ {∥\alpha ∥}_{F}^{2}=1,\\ \alpha \in C^{ND}, \end{array}\right.$$

Tom Tirer and Anthony J. Weiss studied similar programming in [35]. They transformed the cost function into:

(21) $$\left[{\hat{p}}_{e},\hat{\alpha }\right]=\arg\max\tilde{Q}\left(p_{e},\alpha \right)=\frac{1}{\lambda_{\min}\left\{E(p_{e})\right\}},$$

where $\lambda_{\min}\left\{\cdot \right\}$ represents the minimal eigenvalue of a matrix. It is a promotion result of the maximization QP, but it is only a “not bad” solution rather than the optimal one. If Γ(k) is singular, $E(p_{e})$ turns out to be singular, and $\lambda_{\min}\left\{E(p_{e})\right\}=0$. In this case, the cost function reaches a peak. NSP-MUSIC only finds the solutions that make Γ(k) singular rather than true emitter positions.

From another point of view, if ∃i,j, which satisfy $e_{i}\left(p_{e}\right)\approx e_{j}\left(p_{e}\right)$, where $e_{i}\left(p_{e}\right)$ and $e_{j}\left(p_{e}\right)$ are the column *i* and column *j* in $E(p_{e})$, the matrix $E(p_{e})$ turns out to be singular or near singular (It should be noticed that, in a single path positioning application, Γ(k) is a block diagonal matrix, and each block is an M×1 column vector. It is impossible that Γ(k) has the same two columns, but this is possible for a multi-path positioning application. In a multi-path environment, Γ(k) is a block diagonal matrix, and each block is an M×L matrix. It is possible that the block is singular.). In this case, the optimal estimations of path attenuations are $\hat{\alpha }={\left[{\hat{\alpha }}_{1},{\hat{\alpha }}_{2},\ldots ,{\hat{\alpha }}_{\ell \cdot n}\right]}^{T}$, where:

(22) $${\hat{\alpha }}_{z}=\left\{\begin{array}{cc} \frac{\sqrt{2}}{2} & z=i,\\ -\frac{\sqrt{2}}{2} & z=j,\\ 0 & else. \end{array}\right.$$

${\hat{\alpha }}_{z}$ is a feasible solution that satisfies (20). Substitute (22) into (19); $Q(p_{e},\alpha )\to +\infty$. If α are complex scaled path attenuations, the cost function of NSP-MUSIC will reach a peak where $E(p_{e})$ is singular or near singular.

Above all, if the manifold matrix Γ(k) is singular or near singular, SSP-MUSIC will fail to get the emitter positions. Besides, if Γ(k) is singular or near singular and α are complex path attenuations, NSP-MUSIC also fails to get the emitter positions. In the next section, we will discuss the singularity of the manifold matrix Γ(k) and the necessity for non-negative real number constraints for path attenuations.

#### 3.1.3. Singularity of the Manifold Matrix in the Presence of Multi-Path Propagation

We have discussed that SSP-MUSIC and NSP-MUSIC will fail to locate the emitters if Γ(k) is a near singular matrix, and we get the conditions of a candidate that makes Γ(k) near singular in Appendix A.

Unfortunately, Γ(k) will always be near singular. For example, in a shortwave positioning application, the size of a shortwave antenna is large. If the receivers need to be installed on mobile platforms (e.g., aircraft), only one antenna can be installed in a receiver (M=1). In this case, from the condition in Theorem A2 in Appendix A, $a_{\ell_{1}n}\left(k\right)=a_{\ell_{2}n}\left(k\right),k=1,2,\ldots ,K$ always are satisfied, and the manifold matrix Γ(k) of a CSMCis a singular matrix.

In another application, the transponders are installed on a mobile platform (e.g., Unmanned Aerial Vehicle (UAV) platform or satellite platform). If one transponder is relatively close to another transponder, or two transponders and a receiving station are near collinear, this makes $a_{\ell_{1}n}\left(k\right)\approx a_{\ell_{2}n}\left(k\right),k=1,2,\ldots ,K$. In this situation, the conditions in Theorem A2 in Appendix A are satisfied, and Γ(k) turns out to be near singular.

**Figure 2:**
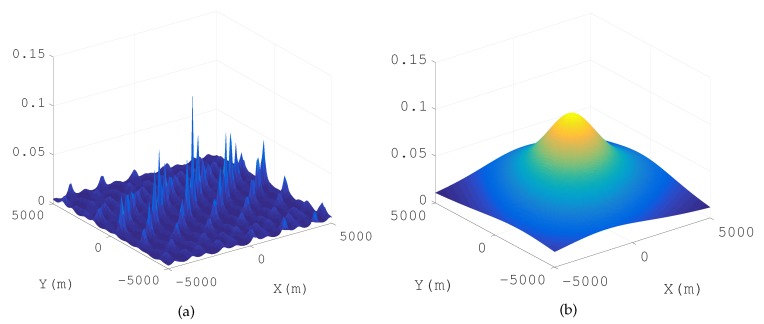
Multiple-peak cost function of a frequency band signal and single peak cost function of a base band signal. (**a**) Cost function for a frequency band signal (**b**) Cost function for a base band signal.

In addition, it is necessary to down convert the Radio Frequency (RF) signals to baseband signals firstly to avoid the multi-peak searching of the cost function (see Figure 2). The suboptimal peaks in Figure 2a are caused by the carrier wave. The higher the frequency of the carrier, the more suboptimal the peaks in the cost function. Besides, there is only one peak in the cost function for a baseband signal positioning model (see Figure 2b). Figure 2b is an up envelopeof Figure 2a. If the cost function is a surface with multiple peaks, it is difficult to develop a searching strategy except for a high density grid searching. However, it is easy to get the global optimal solution for a continuous function with a single peak (e.g., Steepest Descent Method (SDM), Newton Method (NM)). In a baseband signal positioning application, λ≫R, where λ is the wave length corresponding to the maximal frequency of the baseband signal, and *R* is the radius of the circle receiving array. If λ≫R, it is easy to satisfy $a_{\ell_{1}n}\left(k\right)\approx a_{\ell_{2}n}\left(k\right),k=1,2,\ldots ,K$, and Γ(k) is near singular.

#### 3.1.4. Non-Negative Real Path Attenuation Constraints

Our model requires that path attenuations must be non-negative real numbers, but complex values in existing studies for narrow band signal positioning [18,29,31,32,33]. Weiss ignored the path attenuations (set the attenuation α=1) in wide-band emitter positioning in [36].

In a narrow band signal positioning application, it is assumed that $a_{\ell n}\left(k\right)\approx a_{\ell n}\left(k_{0}\right)≜a_{\ell n}$, where k=1,2,⋯,K, and $\omega_{k_{0}}$ is the carrier frequency. Based on the above assumptions, (3) turns out to be:

(23) $$\begin{array}{cc} r_{n}\left(k\right) & =\sum_{\ell =1}^{L}\sum_{d=1}^{D}\alpha_{d\ell n}a_{\ell n}\left(k\right)e^{-i\omega_{k}{\bar{\tau }}_{\ell d}}{\overset{ˇ}{s}}_{d}\left(k\right)+n\left(k\right)\\ & \approx \sum_{\ell =1}^{L}\sum_{d=1}^{D}\alpha_{d\ell n}e^{j\omega_{k_{0}}\tau }a_{\ell n}e^{-i\omega_{k}{\bar{\tau }}_{\ell d}}{\overset{ˇ}{s}}_{d}\left(k\right)+n\left(k\right), \end{array}$$

where $e^{j\omega_{k_{0}}\tau }$ is a phase adjustment item to satisfy $e^{j\omega_{k_{0}}\tau }a_{\ell n}\left(k_{0}\right)\to a_{\ell n}\left(k\right)$. Existing studies used the envelope information only to estimate the propagation delay and dropped the carrier phase information. $e^{j\omega_{k_{0}}\tau }$ was an adjustment factor for carrier phase alignment, and it was used to reduce the interference with the propagation delay estimation. Denote ${\bar{\alpha }}_{d\ell n}≜\alpha_{d\ell n}e^{j\omega_{k_{0}}\tau }$; (23) turns out to be:

(24) $$r_{n}\left(k\right)\approx \sum_{\ell =1}^{L}\sum_{d=1}^{D}{\bar{\alpha }}_{d\ell n}a_{\ell n}e^{-i\omega_{k}{\bar{\tau }}_{\ell d}}{\overset{ˇ}{s}}_{d}\left(k\right)+n\left(k\right),$$

where ${\bar{\alpha }}_{d\ell n}$ is a complex scalar representing the “channel attenuation” (It is not a real channel attenuation coefficient, but an equivalent parameter, which is determined by the real path attenuation and the model error caused by the narrow band signal assumption), and $a_{\ell n}$ denotes the generalized array response matrix.

The commonly-used DPD methods [18] modeled the received signal as:

(25) $$r_{n}\left(k\right)\approx \sum_{d=1}^{D}\alpha_{dn}a_{n}\left(p_{d}\right)e^{-i\omega_{k}\tau_{nd}}{\overset{ˇ}{s}}_{d}\left(k\right)+n\left(k\right).$$

(25) is an LoS positioning model for narrow band signal positioning, while (24) is an NLoS positioning model. Existing models with complex path attenuation assumptions are viewed as simplified models of (3) in a narrow band signal positioning application. We point out that the simplified model (24) can not be adopted either in a MUSIC method or in an ML method in a multi-path propagation and unknown wave form application. We have obtained that the manifold matrix Γ(k) may be singular in Section 3.1.3 and discussed that if the path attenuations were complex numbers, a MUSIC method could not obtain the emitter positions correctly in Section 3.1.2. We will discuss this further in Section 4.1 to explain the necessity of the real and non-negative constraints in an ML method.

Overall, we develop (3) as the signal model for positioning and constrain the path attenuations in $R^{+}$.

### 3.2. Mathematical Model of MP-MUSIC

In an MP-MUSIC method, the optimal estimation of α for a fixed emitter position $p_{e}$ is given by solving the following programming:

(26) $$\begin{array}{c} \hat{\alpha }=\arg\min Q\left(\alpha \right)=\alpha^{H}E\left(p_{e}\right)\alpha , \end{array}$$

$$\begin{array}{c} s.t.\left\{\begin{array}{c} {∥\alpha ∥}_{1}=1,\\ \alpha \in R^{LN},\alpha \geq 0,\\ E\left(p_{e}\right)=\sum_{k=1}^{K}\Gamma^{H}\left(k\right)\left[I_{MN}-U_{s}\left(k\right)U_{s}^{H}\left(k\right)\right]\Gamma \left(k\right). \end{array}\right. \end{array}$$

where ${∥\cdot ∥}_{1}$ is the one norm of the vector (that is, the sum of the absolute values of the elements of the vector) and $I_{MN}$ is an MN×MN identity matrix.

The programming is a non-linear programming with real value constraints. There is an LN dimensional searching for a candidate emitter position, and it is difficult to solve the programming directly. We remove the imaginary items in the programming without changing the optimal solution firstly and prove the convexity of the modified programming later. An iterative algorithm named ASA is proposed to solve the convex programming after the proof.

#### 3.2.1. Remove the Imaginary Items in the Programming

Rewrite the objective function by:

(27) $$\begin{array}{c} \alpha^{H}E\left(p_{e}\right)\alpha =\alpha^{H}\Psi \alpha +\alpha^{H}\Phi \alpha , \end{array}$$

where Ψ≜Re[E(pe)] is the real part of $E(p_{e})$ and Φ≜Im[E(pe)] is the imaginary part of $E(p_{e})$. Φ is a Hermitian matrix, which satisfies:

(28) $$\begin{array}{c} \Phi_{i,j}=\left\{\begin{array}{cc} 0 & i=j,\\ -\Phi_{j,i} & \mathrm{else}. \end{array}\right. \end{array}$$

where $\Phi_{i,j}$ is the element at the *i*-th row and *j*-th column of the matrix Φ. Since α is a non-negative real value vector and the diagonal elements of Φ are zeros,

(29) $$\begin{array}{cc} \alpha^{H}\Phi \alpha & =\sum_{i=1}^{NL}\sum_{j=1}^{NL}\alpha_{i}\Phi_{i,j}\alpha_{j}\\ & =\sum_{i=2}^{NL}\sum_{j=1}^{i-1}\left(\alpha_{i}\Phi_{i,j}\alpha_{j}+\alpha_{j}\Phi_{j,i}\alpha_{i}\right)\\ & =0. \end{array}$$

Substitute (29) into (27), and the cost function turns out to be:

(30) $$\begin{array}{c} \hat{\alpha }=\arg\min q\left(\alpha \right)=\alpha^{H}\Psi \alpha \end{array}$$

$$\begin{array}{c} s.t.\left\{\begin{array}{c} {∥\alpha ∥}_{1}=1,\\ \alpha \in R^{LN},\alpha \geq 0. \end{array}\right. \end{array}$$

The programming (30) is a QP in the real field. If there are no inequality constraints and the objective function is convex, the Lagrange multiplier method is effective for solving the programming. However, the non-negative constraints of path attenuations are necessary due to the singularity of the array manifold. To obtain the optimal solution of (30), we verify the convexity of the programming firstly and design an algorithm to solve the convex programming.

#### 3.2.2. Convexity of the Programming

**Theorem** **1.** (30) *is a convex quadratic programming with linear equality constraints and lower bounds.*

**Proof.**  (31) $$\begin{array}{cc} E(p_{e}) & =\sum_{k=1}^{K}E(k), \end{array}$$

where:

(32) $$\begin{array}{cc} E(k) & =\Gamma^{H}\left(k\right)\left[I_{MN}-U_{s}\left(k\right)U_{s}^{H}\left(k\right)\right]\Gamma \left(k\right)\\ & =\Gamma^{H}\left(k\right)U_{n}\left(k\right)U_{n}^{H}\left(k\right)\Gamma \left(k\right), \end{array}$$

where $U_{n}$ is the noise subspace of the received signal. $\forall x\in R^{LN}$,

(33) $$\begin{array}{cc} x^{H}E\left(p_{e}\right)x & =\sum_{k=1}^{K}x^{H}E\left(k\right)x\\ & =\sum_{k=1}^{K}{∥x^{H}\Gamma^{H}\left(k\right)U_{n}\left(k\right)∥}^{2}\geq 0, \end{array}$$

(34) $$\begin{array}{cc} x^{H}\Psi x & =x^{H}E\left(p_{e}\right)x-x^{H}\Phi x. \end{array}$$

Since $\forall x\in R^{LN},x^{H}\Phi x=0$, and substituting (33) into (34), we get $x^{H}\Psi x\geq 0$, and the objective function is Positive Semi-Definite (PSD). The optimization problem (30) is a convex quadratic programming with linear equality constraints and lower bounds. ☐

It is possible to find the global optimal solution for a convex quadratic programming [37,38]. The interior-point algorithm or any Heuristic Searching Algorithm (HSA) can be adopted to solve the optimization problem with equality constraints and lower bounds. However, those algorithms apply numerical searching strategies with low efficiencies. We introduce a faster algorithm named the Active Set Algorithm (ASA) in this paper to obtain the global optimal solution base on some theoretical analysis.

#### 3.2.3. Active Set Algorithm

The first widely-used algorithm for solving a similar problem is the active set method published by Lawson and Hanson [34,39]. They proposed an active set method to solve the Non-Negative Least Squares (NNLS).

**Definition** **1.** *Active set [40] In mathematical optimization, a problem is defined using an objective function to minimize or maximize and a set of constraints:*

(35) $$g_{1}\left(x\right)\geq 0,\cdots ,g_{k}\left(x\right)\geq 0.$$

*Given a point x in the feasible region, a constraint:*

(36) $$g_{i}\left(x\right)\geq 0,$$

*is called active at x if $g_{i}\left(x\right)=0$ and inactive at x if $g_{i}\left(x\right)>0$. The set of active ones is called the active set and denoted by $A\left(x\right)=\{i|g_{i}\left(x\right)=0\}$.*

We describe the active set method for solving the quadratic programs of the form (30) containing equality and inequality constraints based on the methods described in [34,39].

Denote the optimal solution of (30) by $\hat{\alpha }$. If the active set of the optimal solution $A(\hat{\alpha })$ were known in advance, we could find the optimal solution $\hat{\alpha }$ by applying techniques, such as the Lagrange multiplier method, for equality-constrained QP. The prior knowledge of the active set accelerates the algorithm effectively.

$\alpha^{*}={\left[\alpha_{1}^{*},\alpha_{2}^{*},\ldots ,\alpha_{LN}^{*}\right]}^{T}$ represents the real, but unknown path attenuations. If the *n*-th receiver cannot receive the signal from the emitter, which is reflected by the *ℓ*-th transponder, $\alpha_{\ell n}^{*}=0$, otherwise, $\alpha_{\ell n}^{*}>0$. In most cases, we known the set $\left\{i|\alpha_{i}^{*}=0\right\}$ in advance and have deleted the unconnected path in the model. without loss of generality, we set $\alpha_{i}^{*}>0,i=1,2,\ldots ,LN$. Denote $\hat{\alpha }$ as an estimation of $\alpha^{*}$, that is $\hat{\alpha }\approx \alpha^{*}$. Denote $\hat{\alpha }=\alpha^{*}+\varepsilon$, where ε is the estimation error vector of $\alpha^{*}$. Since $\alpha_{i}^{*}>0$, i=1,2,…,LN, we set the initial work set by $W^{\left(0\right)}=⌀$.

The searching path of the active set algorithm is strictly in the feasible region. Choose a feasible solution as the initial point of the algorithm. Solve the QP with equality constraints in the work set, and get the optimal searching direction. if the searching direction is blocked by some constraints not in the work set, add the constraints, which block the searching path firstly, into the work set. Resolve the new QP with the updated work set, until the searching direction is not blocked by any constraints. The algorithm reaches a local optimum for the current work set. To get an even better solution, we drop one active constraint in the work set to relax the programming. If the objective function cannot be decreased for all constraints in the work set, we get the optimal solution of the original programming. Otherwise, drop the constraint that causes the fastest decrease.

The detail of the ASA is described in Section 1 of the Supplementary File. The spatial spectrum of an emitter is determined by substituting the $\hat{\alpha }$ into the MUSIC cost function (19).

#### 3.2.4. MP-MUSIC Algorithm

The spatial spectrum of the emitter positions requires only a three-dimensional searching, and the size of $E(p_{e})$ is LN×LN, which is usually rather small. The detailed procedure of the MP-MUSIC algorithm is represented in Algorithm 1.   

**Algorithm 1:** MP-MUSIC algorithm.
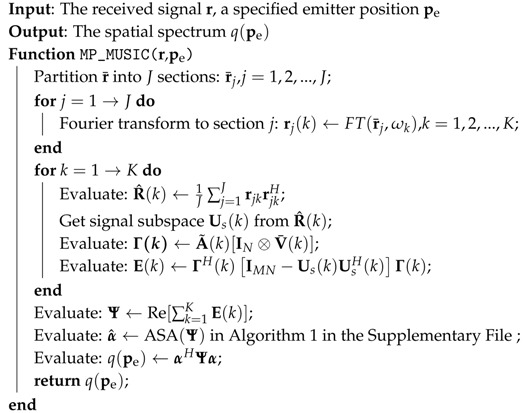


The performance of the MP-MUSIC algorithm is determined by the estimation precision of $\hat{R}\left(k\right)$. If the number of snapshots is not enough, neither the covariance matrix $\hat{R}\left(k\right)$ nor the spatial spectrum $q(p_{e})$ can be estimated precisely.

In a time-sensitive positioning application, it is difficult to get enough snapshots to estimate $\hat{R}\left(k\right)$. We develop a Maximum Likelihood method in the presence of Multi-path Propagation (MP-ML) to estimate the emitter positions directly.

## 4. MP-ML Method

The MP-ML method maximizes the conditional likelihood function of the received signals. The noise is assumed as Additive White Gaussian Noise (AWGN) with a known standard deviation σ,

### 4.1. Mathematical Model of MP-ML

The likelihood function of the received signals is:

(37) $$P\left(r|\theta \right)=\prod_{k=1}^{K}\frac{1}{\left|\pi \Sigma \right|}e^{-{\left[r\left(k\right)-A\left(k\right)\overset{ˇ}{s}\left(k\right)\right]}^{H}\Sigma^{-1}\left[r\left(k\right)-A\left(k\right)\overset{ˇ}{s}\left(k\right)\right]},$$

where r is the observed data, Σ is the covariance matrix of noises, which is defined in (6), and the unknown parameter vector $\theta ≜{\left[p_{e}^{T},\alpha^{T},s^{T}\right]}^{T}$ consists of:

(38) $$\begin{array}{cc} p_{e} & ≜{\left[p_{e}^{T}\left(1\right),p_{e}^{T}\left(2\right),\ldots ,p_{e}^{T}\left(D\right)\right]}^{T},\\ p_{e}\left(d\right) & ≜{\left[p_{ex}\left(d\right),p_{ey}\left(d\right),p_{ez}\left(d\right)\right]}^{T},\\ \bar{\alpha } & ≜{\left[{\bar{\alpha }}_{1}^{T},{\bar{\alpha }}_{2}^{T},\ldots ,{\bar{\alpha }}_{N}^{T}\right]}^{T},\\ {\bar{\alpha }}_{n} & ≜{\left[\alpha_{1n}^{T},\alpha_{2n}^{T},\ldots ,\alpha_{Dn}^{T}\right]}^{T},\\ \overset{ˇ}{s} & ≜{\left[{\overset{ˇ}{s}}^{T}\left(1\right),{\overset{ˇ}{s}}^{T}\left(2\right),\ldots ,{\overset{ˇ}{s}}^{T}\left(K\right)\right]}^{T}, \end{array}$$

where $\alpha_{dn}$ and $\overset{ˇ}{s}\left(k\right)$ have been defined in (5). The log-likelihood function of (37) is:

(39) $$L\left(\theta \right)=-KMN\log\pi \sigma^{2}-\frac{1}{\sigma^{2}}\sum_{k=1}^{K}{\left[r\left(k\right)-A\left(k\right)\overset{ˇ}{s}\left(k\right)\right]}^{H}\left[r\left(k\right)-A\left(k\right)\overset{ˇ}{s}\left(k\right)\right].$$

Remove the constant items, and get the modified cost function of MP-ML:

(40) $$\hat{\theta }=\arg\min\bar{Q}\left(\theta \right)=\sum_{k=1}^{K}{\left[r\left(k\right)-A\left(k\right)\overset{ˇ}{s}\left(k\right)\right]}^{H}\left[r\left(k\right)-A\left(k\right)\overset{ˇ}{s}\left(k\right)\right].$$

The searching space dimension of (40) is 3D+DNL+DK, and it is necessary to reduce the searching space dimension. For fixed attenuations α and emitter position combination $p_{e}$ in (40), the optimal estimation of the source signals at frequency $\omega_{k}$ is:

(41) $$\hat{\overset{ˇ}{s}}\left(k\right)=A^{+}\left(k\right)r\left(k\right),$$

where $A^{+}\left(k\right)≜{\left[A^{H}\left(k\right)A\left(k\right)\right]}^{-1}A^{H}\left(k\right)$ is the Moore–Penrose inverse of A(k). Substitute (41) into (40),

(42) $$\hat{\eta }=\arg\min\bar{Q}\left(\eta \right)=\sum_{k=1}^{K}{\left[r\left(k\right)-P_{A}\left(k\right)r\left(k\right)\right]}^{H}\left[r\left(k\right)-P_{A(k)}r\left(k\right)\right],$$

where $\eta ≜{\left[p_{e}^{T},{\bar{\alpha }}^{T}\right]}^{T}$, $\bar{\alpha }=\alpha I_{D}≜{\left[\alpha_{1,1,1},\cdots ,\alpha_{d,\ell ,n},\cdots ,\alpha_{DLN}\right]}^{T}$, $I_{D}$ is a column vector of *D* ones. $P_{A}\left(k\right)=A\left(k\right)A^{+}\left(k\right)$ is the projection matrix of A(k). Expand (42):

(43) $$\begin{array}{cc} \bar{Q}\left(\eta \right) & =\sum_{k=1}^{K}\left[r^{H}\left(k\right)r\left(k\right)-r^{H}\left(k\right)P_{A}^{H}\left(k\right)r\left(k\right)-r^{H}\left(k\right)P_{A}\left(k\right)r\left(k\right)+r^{H}\left(k\right)P_{A}^{H}\left(k\right)P_{A}\left(k\right)r\left(k\right)\right], \end{array}$$

and move the constant items $r^{H}\left(k\right)r\left(k\right)$. Applying the properties of the projection matrix $P_{A}\left(k\right)=P_{A}^{H}\left(k\right)$ and $P_{A}^{H}\left(k\right)P_{A}\left(k\right)=P_{A}\left(k\right)$, we get the modified programming of MP-ML:

(44) $$Q\left(\eta \right)=-\sum_{k=0}^{K-1}r^{H}\left(k\right)P_{A}\left(k\right)r\left(k\right),$$

$$\begin{array}{c} s.t.\left\{\begin{array}{c} P_{A}\left(k\right)=A\left(k,p_{e}\right)A^{+}\left(k,p_{e}\right),\\ A^{+}\left(k\right)={\left[A^{H}\left(k,p_{e}\right)A\left(k,p_{e}\right)\right]}^{-1}A^{H}\left(k,p_{e}\right),\\ A\left(k,p_{e}\right)=\Gamma \left(k,p_{e}\right)\alpha ,\\ \Gamma \left(k,p_{e}\right)=\tilde{A}\left(k,p_{e}\right)V\left(k,p_{e}\right),\\ \bar{\alpha }=\alpha I_{D},\\ \bar{\alpha }\in R^{DLN},\\ \bar{\alpha }\geq 0. \end{array}\right. \end{array}$$

There are two differences between our model and Bar-Shalom Ofer’s model in [28]. The first one is that only a single emitter and a single receiver were modeled in their work, but multiple emitters and multiple receivers are taken into consideration in our work. The second one is that there are complex path attenuations in [28], but real non-negative path attenuations in our model.

The cost function in [28] was modeled as:

(45) $$\max Q\left(\eta \right)=-\sum_{k=1}^{K}\frac{\alpha^{H}f\left(k\right)f^{H}\left(k\right)\alpha }{\alpha^{H}C\left(k\right)\alpha }.$$

where $f\left(k\right)≜\Gamma^{H}\left(k,p_{e}\right)r\left(k\right)$, $C\left(k\right)≜\Gamma^{H}\left(k,p_{e}\right)\Gamma \left(k,p_{e}\right)$. Section 3.1.1 has discussed that $\Gamma (k,p_{e})$ may be singular, and C(k) may be singular, as well. When C(k) is singular, there is an α that satisfies $\alpha^{H}C\left(k\right)\alpha =0$, and the cost function Q(η) reaches the peak. However, the candidate emitter position is not the true emitter position. If $\Gamma (k,p_{e})$ is near singular, $f\left(k\right)=\Gamma^{H}\left(k,p_{e}\right)r\left(k\right)$ is near singular, as well. The numerator and denominator of the cost function Q(η) both tend to zero, and the value of the cost function turns out to be unstable. The noise level will seriously affect the value of the cost function in this case, and the model cannot find the emitter accurately.

The searching space dimension of (44) has been reduced to 3D+DNL, but it is still difficult to solve such a high dimensional non-linear programming. We propose an iterative algorithm in this paper to get the estimation of path attenuations to reduce the time consumption of the MP-ML.

### 4.2. Remove Imaginary Items in the Programming

Substitute the constraints into the objective function of (44),

(46) $$\begin{array}{cc} Q(\eta ) & =-\sum_{k=1}^{K}r^{H}\left(k\right)\Gamma \left(k,p_{e}\right)\alpha {\left[\alpha^{H}\Gamma^{H}\left(k,p_{e}\right)\Gamma \left(k\right)\alpha \right]}^{-1}\alpha^{H}\Gamma^{H}\left(k,p_{e}\right)r\left(k\right)\\ & =-\sum_{k=1}^{K}f^{H}\left(k\right)\alpha {\left[\alpha^{H}C\left(k\right)\alpha \right]}^{-1}\alpha^{H}f\left(k\right), \end{array}$$

where $f\left(k\right)≜\Gamma^{H}\left(k,p_{e}\right)r\left(k\right)$, $C\left(k\right)≜\Gamma^{H}\left(k,p_{e}\right)\Gamma \left(k,p_{e}\right)$.

Henk A. L. Kiers studied a similar convex optimization problem in [41]. Ofer Bar-Shalom and Anthony J. Weiss study the complexity form of the optimization in [28] and its application in [29]. The programming in our work has the complex f(k) and C(k), but the decision making variables α are real non-negative values. We modify the iterative process in [28,41] to satisfy the real non-negative constraints in our work.

The cost function (46) can be rewritten by:

(47) $$\begin{array}{cc} Q(\eta ) & =-\sum_{k=0}^{K-1}f^{H}\left(k\right)\alpha {\left[\alpha^{H}C\left(k\right)\alpha \right]}^{-1}\alpha^{H}f\left(k\right)\\ & =-\sum_{k=0}^{K-1}\mathrm{tr}\left\{\alpha^{H}f\left(k\right)f^{H}\left(k\right)\alpha {\left[\alpha^{H}C\left(k\right)\alpha \right]}^{-1}\right\}, \end{array}$$

where tr(·) is the trace operator of a matrix. Since C(k) and $f\left(k\right)f^{H}\left(k\right)$ are Hermitian metrics, ∀α satisfy:

(48) $$\begin{array}{cc} \alpha^{H}C\left(k\right)\alpha & =\alpha^{H}\bar{C}\left(k\right)\alpha ,\\ \alpha^{H}f\left(k\right)f^{H}\left(k\right)\alpha & =\alpha^{H}\bar{f}\left(k\right){\bar{f}}^{H}\left(k\right)\alpha , \end{array}$$

where C¯(k)≜Re{C(k)}, $\bar{f}\left(k\right)=u\left(k\right)s^{\frac{1}{2}}\left(k\right)$, and $u\left(k\right)s\left(k\right)v^{H}\left(k\right)$ is the SVD decomposition of Re{f(k)fH(k)}. The complex matrices f(k) and C(k) are replaced by the matrices $\bar{f}\left(k\right)$ and $\bar{C}\left(k\right)$ with real number elements:

(49) $$\begin{array}{cc} Q(\eta ) & =-\sum_{k=1}^{K}\mathrm{tr}\left\{\alpha^{H}f\left(k\right)f^{H}\left(k\right)\alpha {\left[\alpha^{H}C\left(k\right)\alpha \right]}^{-1}\right\}\\ & =-\sum_{k=1}^{K}{\bar{f}}^{H}\left(k\right)\alpha {\left[\alpha^{H}\bar{C}\left(k\right)\alpha \right]}^{-1}\alpha^{H}\bar{f}\left(k\right). \end{array}$$

The complex non-linear programming with real constraints (46) is simplified to be a real non-linear programming (49).

### 4.3. An Iterative Algorithm for Solving MP-ML

We introduce a theorem firstly and then give an iterative algorithm for solving the programming (49).

**Theorem** **2.** *${\bar{\alpha }}_{i}$ is a feasible solution of* (49)*, and a better solution of* (49) *is obtained by solving the following programming:*

(50) $${\bar{\alpha }}_{i+1}=\arg\min_{\bar{\alpha }}G\left(\alpha_{i},\bar{\alpha },p_{e}\right)=||Y-{\bar{\alpha }}^{H}X{\left|\right|}^{2}-Z,$$

$$\begin{array}{c} s.t.\;\bar{\alpha }\geq 0. \end{array}$$

*where:*

$$\begin{array}{cc} F & ≜\sum_{k=1}^{K}\bar{f}{\left(k\right)}^{T}W{\left(k\right)}^{T},\\ Y & ≜{\mathrm{FU}}^{-1},\\ X & ≜U^{T},\\ Z & ≜{\mathrm{YY}}^{T},\\ U & =\bar{U}\Sigma^{\frac{1}{2}},\\ W(k) & ≜I_{N}⊗\mathrm{diag}\left\{w\left(k\right)\right\}⊗I_{L},\\ w(k) & ≜\bar{f}{\left(k\right)}^{T}\alpha_{i}{\left[\alpha_{i}^{T}\bar{C}\left(k\right)\alpha_{i}\right]}^{-1}, \end{array}$$

*$I_{L}$ is an identify matrix with a size of L×L and $I_{N}$ is an identify matrix with a size of N×N. $\bar{U}$ is the Singular Value Decomposition (SVD) of the following item:*

$$\begin{array}{cc} \sum_{k=1}^{K}W\left(k\right)\bar{C}\left(k\right)W^{T}\left(k\right) & ={\bar{U}}^{T}\Sigma \bar{U}. \end{array}$$

The proof of Theorem 2 is given in Appendix B. The programming (50) is a linear least squares with bound constraints, and the Trust-Region-Reflective (TRR) algorithm is adopted to solve the programming. The detail of TRR is described in [42,43,44].

Following Theorem 2 and (A11) in the Appendix B:

(51) $$Q\left(\eta_{i+1}\right)=H\left({\bar{\alpha }}_{i+1}\right)\leq G\left(\alpha_{i},{\bar{\alpha }}_{i+1},p_{e}\right)\leq G\left(\alpha_{i},{\bar{\alpha }}_{i},p_{e}\right)=H\left({\bar{\alpha }}_{i}\right)=Q\left(\eta_{i}\right).$$

We get an iterative method to obtain a better solution than the previous step. Denote the initial estimation of the unknown channel attenuations by $\alpha_{0}$. The solution of (50) gives a better estimation of the unknown parameter vector due to the inequality of (51). Furthermore, we get a better estimation ${\bar{\alpha }}_{2}$, so that $H\left({\bar{\alpha }}_{2}\right)\leq G\left(\alpha_{1},{\bar{\alpha }}_{2},p_{e}\right)\leq G\left(\alpha_{1},{\bar{\alpha }}_{1},p_{e}\right)=H\left({\bar{\alpha }}_{1}\right)$. Thus, $H\left({\bar{\alpha }}_{2}\right)\leq H\left({\bar{\alpha }}_{1}\right)\leq H\left({\bar{\alpha }}_{0}\right)$. The detail of the iterative procedure is shown in the Algorithm 2 in the supplementary file.

### 4.4. Getting the Initial Value

The performance of the iterative algorithm is determined by the initial value ${\bar{\alpha }}_{0}$. The path attenuations from the MP-MUSIC algorithm are used as the initial value of the MP-ML.

The initial path attenuations of the emitter *d* are denoted by ${\bar{\alpha }}_{0}\left(d\right)$, and they are estimated by the MP-MUSIC in Algorithm 1.

For a fixed emitter position combination $p_{e}$, evaluate the MP-MUSIC algorithm to get the initial path attenuations ${\hat{\alpha }}_{d}$ of the position $p_{e}\left(d\right)$, d=1,2,…,D.

Reshape $\hat{\alpha }={\left[{\hat{\alpha }}_{1}^{T},{\hat{\alpha }}_{2}^{T},,\ldots ,{\hat{\alpha }}_{D}^{T}\right]}^{T}$ to get the initial attenuations vector:

(52) $${\bar{\alpha }}_{0}={\left[{\bar{\alpha }}_{1}^{T},{\bar{\alpha }}_{2}^{T},\ldots ,{\bar{\alpha }}_{D}^{T}\right]}^{T}$$

where:

$$\begin{array}{cc} {\hat{\alpha }}_{d} & ={\left[{\hat{\alpha }}_{d1}^{T},{\hat{\alpha }}_{d2}^{T},\ldots ,{\hat{\alpha }}_{dN}^{T}\right]}^{T},\\ {\hat{\alpha }}_{dn} & ={\left[{\hat{\alpha }}_{dn1},{\hat{\alpha }}_{dn2},,\ldots ,{\hat{\alpha }}_{dnL}\right]}^{T}, \end{array}$$

and ${\hat{\alpha }}_{dn\ell }$ is estimated from MP-MUSIC.

### 4.5. MP-ML Algorithm

Algorithm 2 in the Supplementary File optimizes the parameters α for a fixed emitter positions combination $p_{e}$. The searching dimension is reduced to 3D further. It is possible to solve a 3D dimensional nonlinear programming. The MP-ML algorithm is proposed in Algorithm 2

Define the Region of Interest (RoI) by p, and apply the MP-MUSIC algorithm described in Algorithm 1 to get an initial solution $p_{e}={\left[p_{e}^{T}\left(1\right),p_{e}^{T}\left(2\right),\ldots ,p_{e}^{T}\left(D\right)\right]}^{T}$ and the corresponding path attenuations $\alpha_{0}$. Adopt Algorithm 2 in the Supplementary File to get the optimal estimations of attenuations α and the cost function of emitter positions $p_{e}$. Design a suitable searching path $p_{e}^{i}$, i=1,2,…, such as the Gaussian method, and get the optimal emitter position estimations.

**Algorithm 2:** MP-ML algorithm.
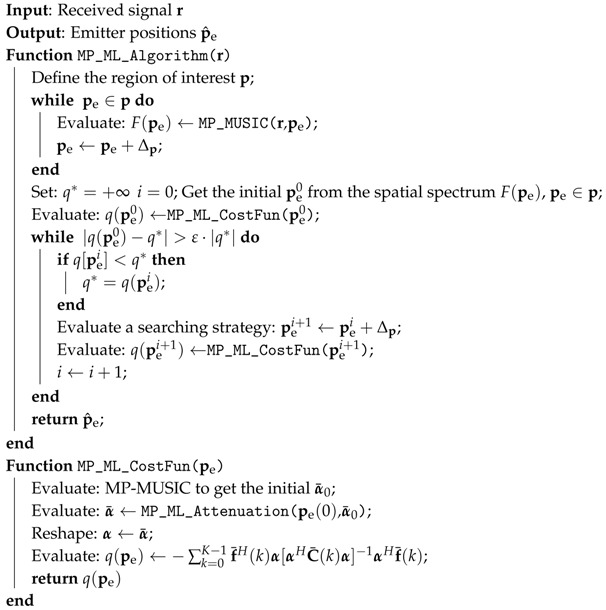


## 5. Numerical Examples

Some numerical examples are given to demonstrate the performances of the above algorithms.

### 5.1. Scenario Setting and Performance Index Definition

**Figure 3:**
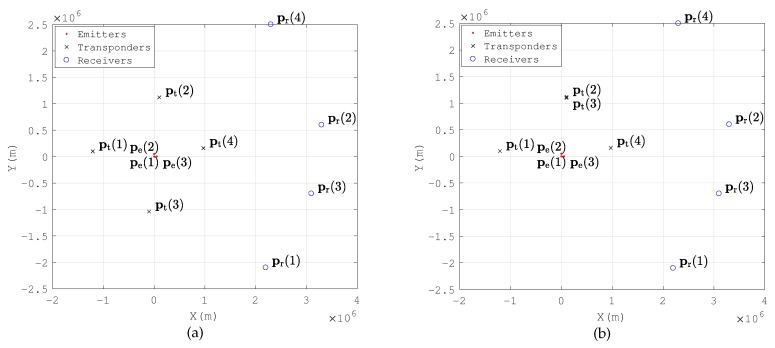
Layouts of the numerical examples. (**a**) Layout A (**b**) Layout B.

In numerical simulations, three emitters are located at [0,0,0], [50,0,0] and [0,50,0], and four receiving arrays are located at [2200,−2100,0], [3300,600,0], [3100,−700,0] and [2300,2500,0]. There are two different layouts of transponders in the simulations. The first one is the basic scenario, and the second one is designed to test the performances when a transponder is close to the anther. There are four transponders located at [−1210,100,200],[100,1120,200], (−100,−1040,200) and [970,160,200] in Scenario A (see Figure 3a) (Figure 3 is a top view of the system layout. Height information is not indicated in the figure.). We move the third transponder to [100,1100,200] in Scenario B (see Figure 3b). All the positions are measured in km.

Each receiving array is a Uniform Circular Array (UCA) with eleven antennas and a radius of 30 m. The bandwidth is 8 kHz. The carrier frequency is 10 MHz. The simulation results are based on 200 Monte Carlo runs to gather enough statistics. The source signal of each emitter and the path attenuation coefficients are generated randomly once for all the Monte Carlo runs, while the additive noises are regenerated at each run. The complex-valued signal frequency coefficients are subject to ${∥{\overset{ˇ}{s}}_{d}∥}_{F}^{2}=1$. The path attenuation coefficients are drawn from a uniform distribution between zero and one. The SNR is defined in terms of “post-processing SNR”, which is given by:

(53) $$\begin{array}{cc} \mathrm{SNR} & ≜\frac{E\left\{\sum_{k=1}^{K}{∥A\left(k\right)\overset{ˇ}{s}\left(k\right)∥}_{F}^{2}\right\}}{K\sigma^{2}}. \end{array}$$

Root-Mean-Squared Error (RMSE) of the estimated position is adopted as the performance index of the algorithms. RMSE is given by:

(54) $$\mathrm{RMSE}\left(p_{e}\right)≜\sqrt{\frac{\sum_{i=1}^{N_{s}}{∥{\hat{p}}_{e}-p_{e}∥}_{F}^{2}}{N_{s}D}},$$

where $N_{s}$ is the number of Monte Carlo runs, *D* is the number of emitters, $p_{e}$ are the real emitter positions and ${\hat{p}}_{e}$ are the estimated emitter positions

A scalar quantity of the CRLB matrix corresponding to RMSE is defined as:

(55) $$\bar{\mathrm{CRLB}}\left(p_{e}\right)≜\sqrt{\frac{\lambda_{\max}\left[\mathrm{CRLB}\left(p_{e}\right)\right]}{D}},$$

where $\lambda_{\max}\left[\mathrm{CRLB}\left(p_{e}\right)\right]$ is the maximal eigenvalue of $\mathrm{CRLB}(p_{e})$. The computation of the CRLB matrix is presented in Section 3 of the Supplementary File [45,46,47].

### 5.2. Performances of the MP-MUSIC Method

We compare the performances of the following MUSIC algorithms:

- SSP-MUSIC: Signal Subspace Projection MUSIC proposed in [18],
- NSP-MUSIC: Noise Subspace Projection MUSIC without non-negative and real constraints,
- MP-MUSIC-IPA: Noise subspace projection MUSIC with real and non-negative constraints in the multipath propagation scenario and solved by the Interior Point Algorithm,
- MP-MUSIC-ASA: Noise subspace projection MUSIC with real and non-negative constraints in the multipath propagation scenario and solved by the Active Set Algorithm.

#### 5.2.1. SSP-MUSIC and NSP-MUSIC in a Single Path Propagation Positioning Scenario

**Figure 4:**
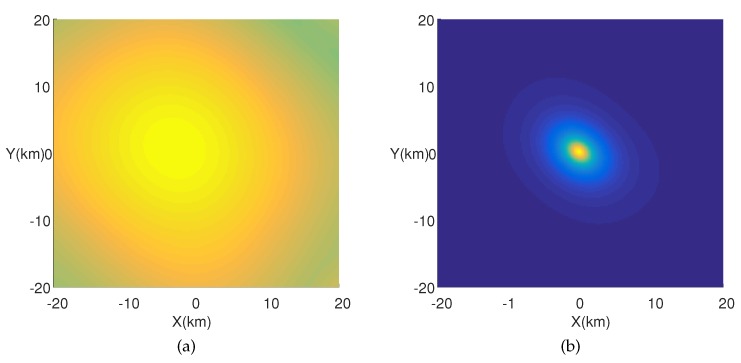
Spatial spectrum of Signal Subspace Projection (SSP)-MUSIC and Noise Subspace Projection (NSP)-MUSIC in a single path scenario. (**a**) SSP-MUSIC (**b**) NSP-MUSIC.

The single emitter is placed at [0,0] (km), and only the direct paths are taken into consideration. Figure 4 gives the spatial spectrum in a single path propagation positioning scenario (Actually, the spatial spectrum of a 3D positioning is a 3D spectrum, but in order to show the form of spatial spectrum more intuitively, the spatial spectrum displayed in this paper only gives a horizontal slice of the 3D spatial spectrum at the real *Z* value (where z=0)).

We find the peak in the spatial spectrum of SSP-MUSIC (Figure 4a), but the NSP-MUSIC proposed in this paper (Figure 4b) holds a sharper peak and more precise estimation than SSP-MUSIC in a single path positioning context.

The following simulations are designed to study the performance of those MUSIC methods in the presence of multi-path propagation.

#### 5.2.2. SSP-MUSIC, NSP-MUSIC and MP-MUSIC in a Multi-Path Propagation Positioning

We design four numerical simulations in this section. The simulation parameters are set as in Table 1, where *R* is the radius of the UCA, λ and *f* are the carrier wave length and frequency, *M* is the number of the antennas in a receiving array, *B* is the bandwidth of the source signals, *K* is the number of frequencies and *J* is the number of sections of a received signal.

**Figure 5:**
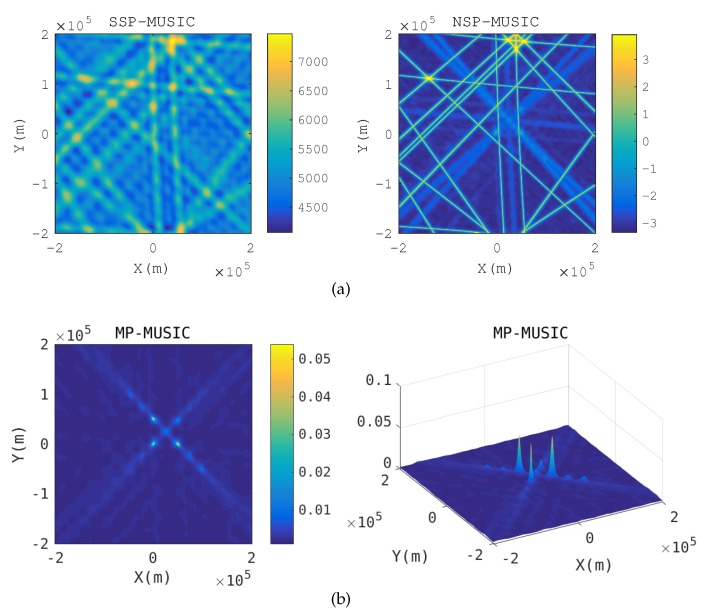
Spatial spectrum in baseband signal positioning. (**a**) Spatial spectrum of SSP-MUSIC and NSP-MUSIC (**b**) Spatial spectrum of Multi-path Propagation (MP)-MUSIC.

Baseband signal positioning: Figure 5 gives the spatial spectrum when R≪λ. The emitter positions $p_{e}$ that make $E(p_{e})$ singular or nearly singular constitute the yellow hyperbolic curves in the SSP-MUSIC spectrum and the NSP-MUSIC spectrum. Neither SSP-MUSIC nor NSP-MUSIC find the emitters correctly when R≪λ. We cannot find any peak in SSP-MUSIC. Three peaks are found in the spectrum of NSP-MUSIC, but they are disrupted by the hyperbolic curves. MP-MUSIC with real and non-negative constraints finds three sharp peaks correctly.

**Figure 6:**
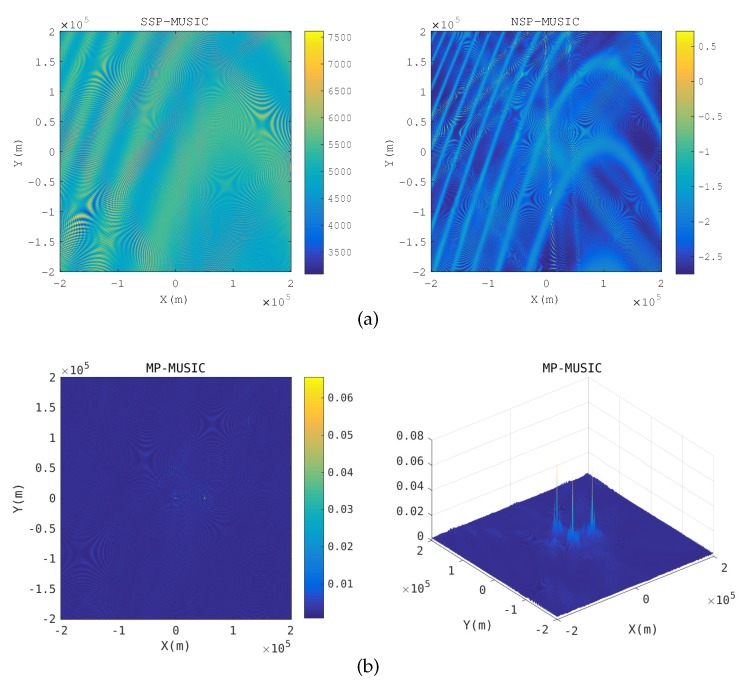
Spatial spectrum when a transponder is close to the anther. (**a**) Spatial spectrum of SSP-MUSIC and NSP-MUSIC (**b**) Spatial spectrum of MP-MUSIC.

Two transponders are close: Figure 6 gives the spatial spectrum when a transponder is close to the anther. The nearest distance of transponders is 20 km in the simulation. When two transponders are close, the array responses of the receiving array with respect to the two transponders are almost same. The yellow hyperbolic curves in the SSP-MUSIC and the NSP-MUSIC spectrum are the candidate positions, which make Γ(k) singular. SSP-MUSIC and NSP-MUSIC failed to locate the emitters in this context, but MP-MUSIC gets the emitter positions correctly.

**Figure 7:**
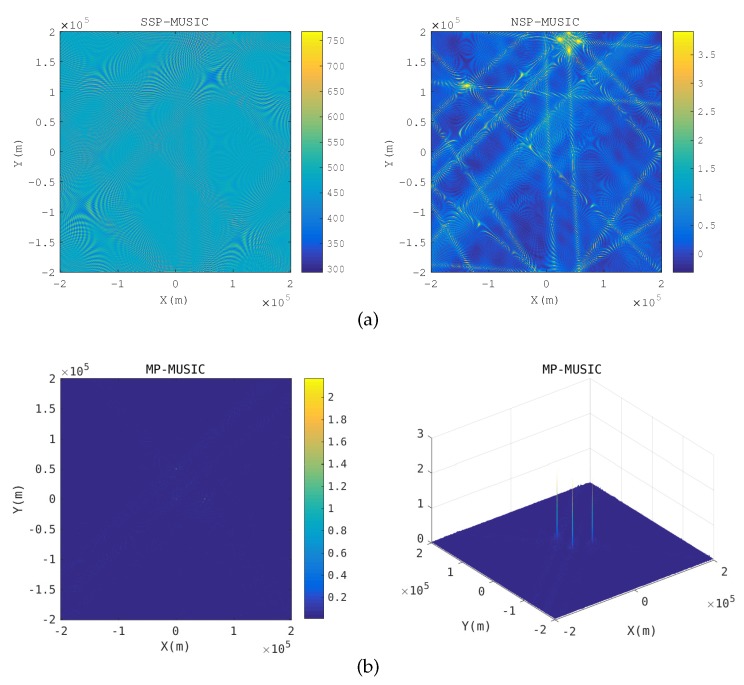
Spatial spectrum for a single antenna of each receiving array. (**a**) Spatial spectrum of SSP-MUSIC and NSP-MUSIC (**b**) Spatial spectrum of MP-MUSIC.

Single antenna of each receiving station: Figure 7 gives the spatial spectrum when M=1. The array responders are defined as $a_{\ell n}\left(k\right)=1$, and an SMC will make Γ(k) singular.

**Figure 8:**
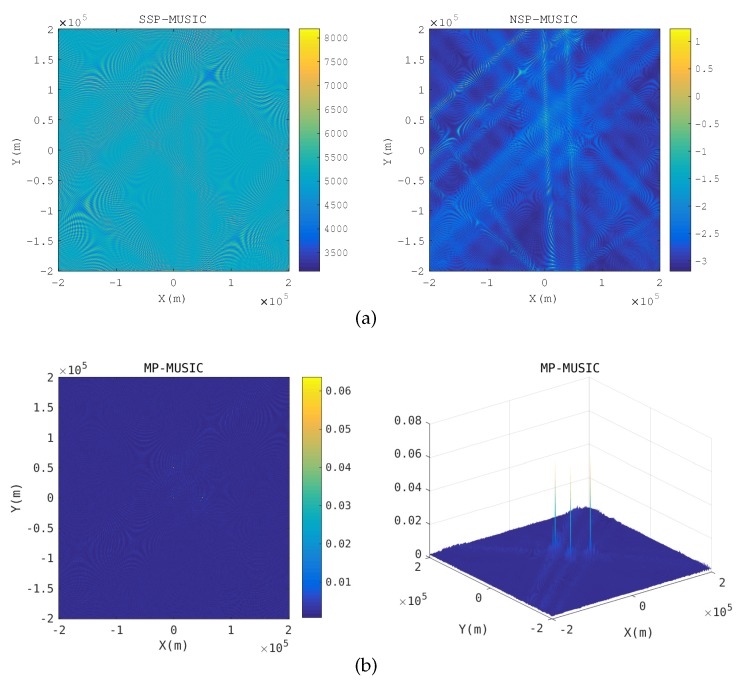
Spatial spectrum in a general scenario. (**a**) Spatial spectrum of SSP-MUSIC and NSP-MUSIC (**b**) Spatial spectrum of MP-MUSIC.

General scenario: Figure 8 gives the spatial spectrum of a general parameter setting scenario. Although there is no deliberate construction of conditions that leads to Γ(k) singularity in the general scenario, SSP-MUSIC and NSP-MUSIC still cannot obtain the emitter positions. However, MP-MUSIC obtains the three emitter locations accurately.

#### 5.2.3. Performances of MP-MUSIC-ASA and MP-MUSIC-IPA

We down convert the radio frequency signals to baseband signals firstly to avoid the multiple peak searching of the radio frequency signal positioning. The simulation parameters are set as in Table 2.

**Figure 9:**
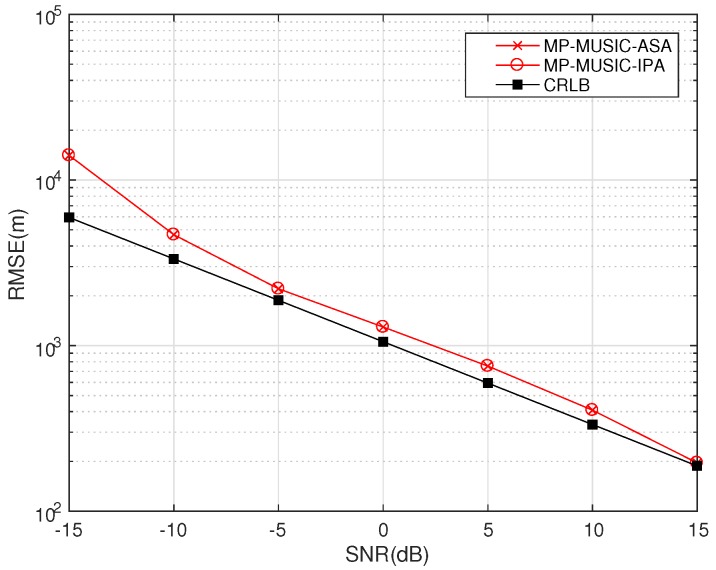
Performance of MP-MUSIC-Active Set Algorithm (ASA) and MP-MUSIC-Interior Point Algorithm (IPA).

The RMSE of MP-MUSIC-ASA, MP-MUSIC-IPA and the CRLB are given in Figure 9.

Since ASA could find the global optimal solution, the performance of MP-MUSIC-ASA should be no worse than that of MP-MUSIC-IPA. The numerical simulation results show that both MP-MUSIC-ASA and MP-MUSIC-IPA find the global optimal solution and have the same RMSE because of the convexity of the cost function.

Simulations are done on a server with Intel Xeon CPU E5-2630 v4, 16 G memory, and Matlab2016a. The MATLAB function “lsqlin” is adopted to verify the performance of IPA. We repeat the simulation 1000 times to get the distribution of the time-consumption of the ASA and IPA sections. It is assumed that the time consumptions $t_{ASA}$ and $t_{IPA}$ follow normal distributions, and $t_{ASA}∼N(5.8175\times 10^{-5}$, $4.8311\times 10^{-6})$, $t_{IPA}∼N\left(3.4756\times 10^{-3},2.2225\times 10^{-5}\right)$ (seconds).

Benefiting from the convex properties and a reasonable initial value of α in ASA, ASA consumes only 1.67% more time than IPA and has a more stable time consumption than IPA (It should be noticed that the time consumption of MP-MUSIC-ASA will be determined by the path attenuations and SNR. A low SNR and small $\alpha_{\ell n}$ make the constraint $\alpha_{\ell n}\geq 0$ an active constraint ($\alpha_{\ell n}=0$.). The time consumption of MP-MUSIC-ASA is deeply affected by the number of elements in the active set.

### 5.3. The Performance of MP-ML and MP-MUSIC

We compare the performances of MP-ML and MP-MUSIC in cases of different numbers of snapshots.

#### 5.3.1. Insufficient Snapshots

If the number of snapshots is insufficient, we cannot obtain a reliable covariance matrix estimation of observations, and MP-MUSIC will fail to get the emitter positions. The simulation parameters are set as in Table 3.

**Figure 10:**
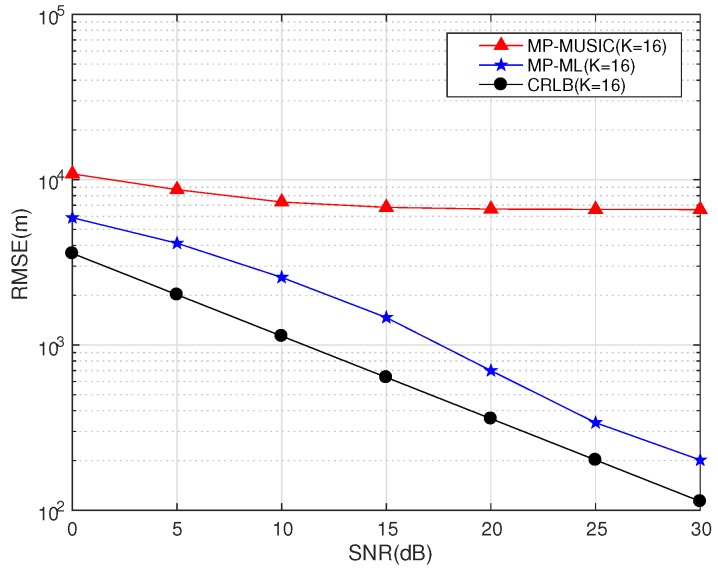
Performances of MP-MUSIC and MP-ML (K=16,J=1).

The performances of MP-MUSIC and MP-ML with 32 snapshots (16 frequencies) are compared in Figure 10.

Only 32 snapshots of each receiver are taken for positioning. Thirty two snapshots are not divided into sections to estimate the covariance matrix in NSP-MUSIC (that is K=16,J=1). The RMSE of NSP-MUSIC cannot be reduced further with the increasing of SNR because of the error of the covariance matrix estimation. The MP-ML obtains a better performance than the MP-MUSIC in the sense of insufficient snapshots.

#### 5.3.2. Performances of Different *K* and *J* Combinations

**Figure 11:**
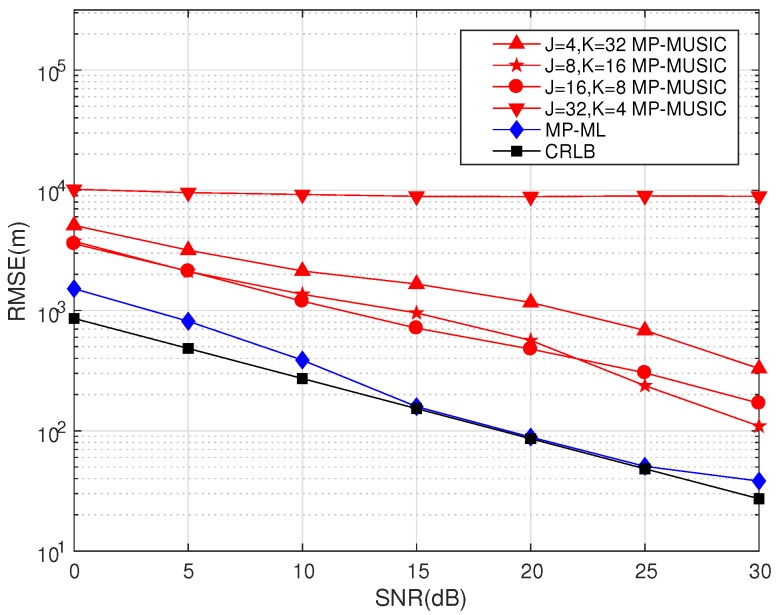
Performance of MP-ML and MP-MUSIC with different J,K combinations.

We discuss the performances of MP-MUSIC with different *K* and *J* combinations in Figure 11. The total number of snapshots in the simulations is 256 (K·J=128). The other parameters are set as in Table 4.

Figure 11 gives the performances of MP-MUSIC with different combinations of *J* and *K*. The number of the snapshots used in the simulation is 256. MP-ML establishes a maximum likelihood function of the 256 snapshots to estimate the emitter positions together, and it has a better performance than MP-MUSIC.

The performances of MP-MUSIC methods are effected by the error of covariance estimations and the bandwidth. A smaller *J* leads to a larger error of the covariance estimation; in addition, a smaller *K* leads to a smaller number of observation equations.

#### 5.3.3. The Performances of Different Numbers of Snapshots

**Figure 12:**
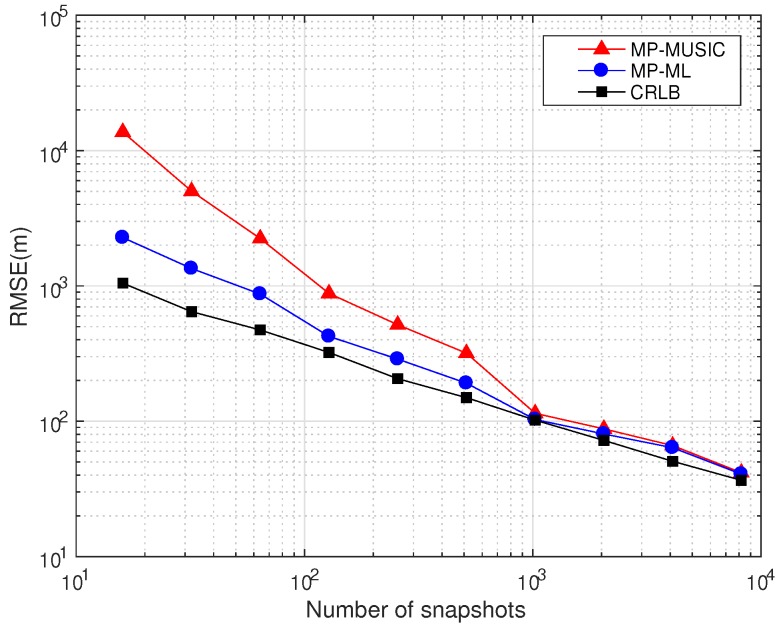
Performances of MP-MUSIC and MP-ML with different numbers of snapshots.

We compare the performances of the MP-MUSIC and the MP-ML with different numbers of snapshots in Figure 12 (The CRLB derived in the Appendix shows that the CRLB is determined by the signal snapshots. The CRLB in Figure 12 is the average of 100 simulations with random signal snapshots.). The other parameters are set as in Table 5.

The performances of MP-MUSIC and MP-ML with $K\cdot J=2^{i}$, (i=4,5,⋯,13) are studied in this section. Since the performance of MP-MUSIC is determined by the combination of *K* and *J*, we choose the best combination of K,J in the MP-MUSIC simulation. As the number of snapshots increases, the RMSE decreases gradually in Figure 12. If the number of snapshots is abundant (K·J>1024), both MP-MUSIC and MP-ML are close to the CRLB. If the number of snapshots is insufficient (K·J<256), the performance of MP-MUSIC will suffer a serious deterioration. However, MP-ML is much less affected by the insufficient snapshots.

#### 5.3.4. Time Consumptions of MP-MUSIC and MP-ML

**Figure 13:**
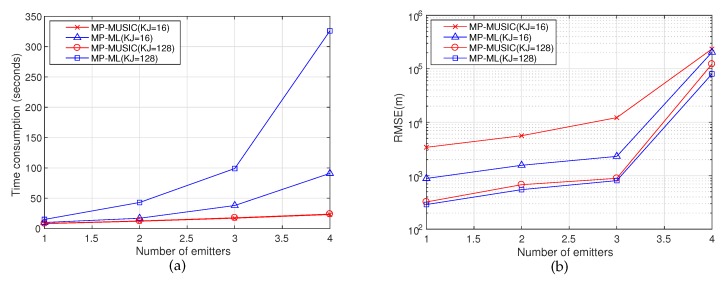
Time consumptions and RMSE of different numbers of emitters. (**a**) Time consumptions of MP-MUSIC and MP-ML (**b**) RMSE of MP-MUSIC and MP-ML.

MP-MUSIC computes the cost values of the candidate one by one to obtain the spatial spectrum; while MP-ML computes the likelihood function of all combinations of *d* emitters. MP-ML consumes more time to obtain the emitter positions than MP-MUSIC. We compare the time consumptions and the corresponding performances of MP-MUSIC and MP-ML with different *d*. The simulation parameters are set as in Table 6, and the simulation results are given in Figure 13.

The layout of transponders and receiving arrays are defined as in Figure 3a, and the four emitters are placed at [0,0,0],[50,0,0],[0,50,0],[50,50,0] (km). We choose *d* emitters in four random placements to analyze the time consumptions of MP-MUSIC and MP-ML with different *d*. We perform 100 simulation runs to gather enough statistics.

In Figure 13a, because MP-MUSIC calculates the cost function value of each candidate one by one, while MP-ML computes all combinations of *d* emitters, the time consumption of MP-MUSIC is almost independent of *d*, and MP-ML increases exponentially with *d*. The time consumption of MP-MUSIC is mainly determined by *K* rather than the number of snapshots (the two red lines in Figure 13a, which represent MP-MUSIC (K=16,J=8) and MP-MUSIC (K=16,J=1), are almost coincident), because the dimension of the matrix in the cost function is determined by *K*, and it is independent of *J*. Besides, the time consumption of MP-ML is determined by the number of snapshots (KJ=128 costs much more time than KJ=16).

Figure 13b presents the performances corresponding to Figure 13a. When the snapshots are sufficient (KJ=128), the positioning accuracy improvement of MP-ML is not significant compared to MP-MUSIC, but it costs much more time to obtain the positions. In this case, applying MP-MUSIC to get emitter positions is a wise choice. When the snapshots are insufficient (JK=16), MP-ML obtains a better performance than MP-MUSIC, although MP-ML costs much more time to get the results. It is worth much more time to obtain a much better performance when the snapshots are insufficient.

**Figure 14:**
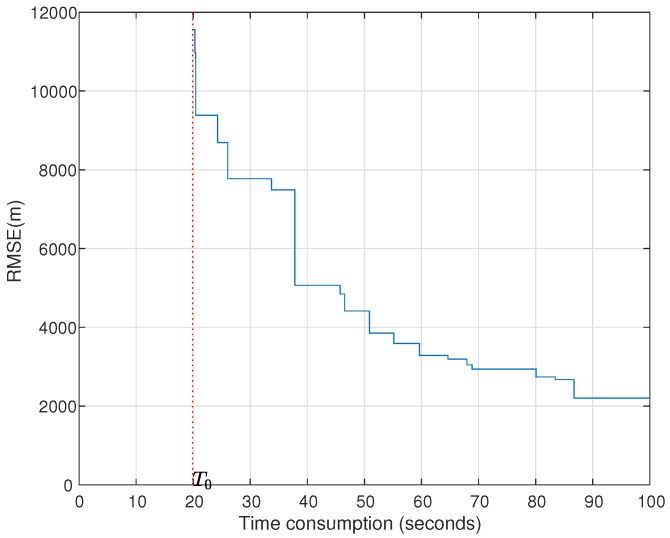
Positioning accuracies and time consumptions of MP-ML.

Benefiting from a good initial value of MP-MUSIC and an efficient searching strategy (steepest descent method), the time consumption of MP-ML is acceptable when the snapshots are insufficient. The time consumption of ASA in MP-MUSIC is determined by the a priori information of the initial active set W, and it can be derived with some other techniques, e.g., direction finding. MP-ML costs much more time than MP-MUSIC, especially in the the case of a large number of emitters. Fortunately, MP-MUSIC, which is adopted to obtain an initial value of MP-ML, provides “not bad” solutions quickly, and the iterative algorithm continues outputting better and better solutions (see Figure 14).

The simulation parameters are set the same as the second row in Table 6, and d=3. MP-ML does not output the result until $T_{0}$, because MP-MUSIC is running from zero to $T_{0}$ to obtain the initial solution of MP-ML. The RMSE of MP-ML is monotone decreasing and continuous for the output results. In real applications, we can find a trade-off between the time consumption and positioning accuracy to obtain an acceptable solution.

### 5.4. Performance of SGP and MP-ML

Single Platform Geolocation (SPG) mentioned in [28] used only one platform to position the single emitter; while MP-ML uses multiple receiving arrays and locates multiple emitters simultaneously.

#### 5.4.1. SPG and MP-ML with a Single Emitter

**Figure 15:**
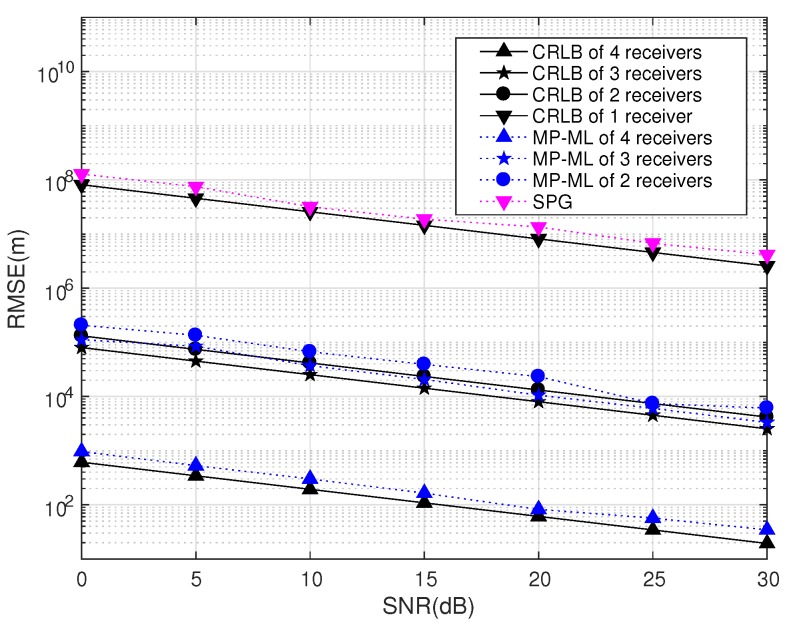
MP-ML and CRLB of different numbers of receivers (K=1024).

Assume that there is only one emitter (D=1, $p_{e}={\left[0,0,0\right]}^{T}$km) in the RoI. Figure 15 compares the performance of SPG and that of MP-ML with different numbers of receivers. The other parameters are set as in Table 7.

We compare the performances of MP-ML with *N* receivers, N=1,2,3,4, and give the corresponding CRLB, as well. When D=1 and N=1, MP-ML degenerates into SPG. The RMSE of *N* receivers is obtained from the average of the RMSE of all combinations of *N* receivers in four positions, and the positions of the four receiving stations are defined as Layout A. The simulation demonstrates that the performance is significantly improved as the number of receiving stations increases. The program with four receiving stations gains nearly a $10^{5}$ performance improvement compared to SPG.

#### 5.4.2. Performance of Positioning Multiple Emitters

MP-ML has the ability of positioning multiple emitters synchronously. We analyze the performances of different numbers of emitters in this section.

**Figure 16:**
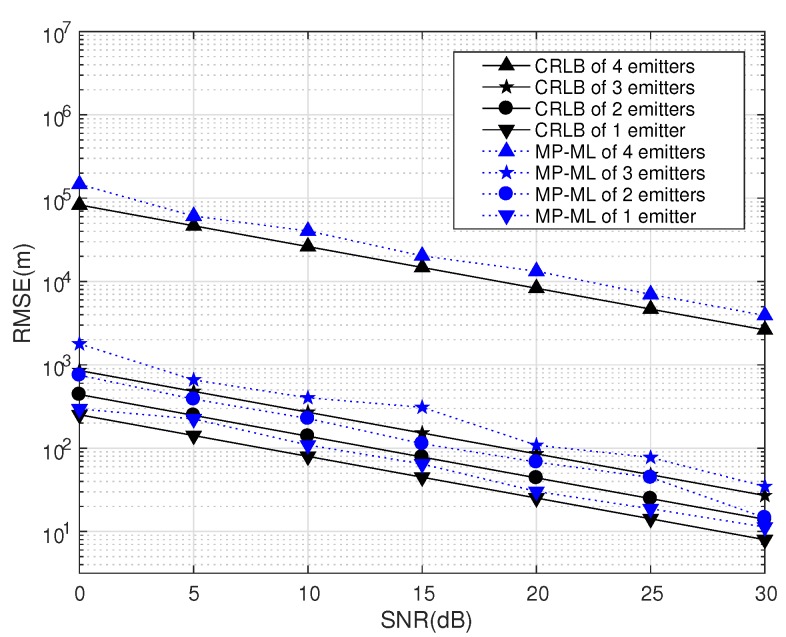
MP-ML and CRLB of different numbers of emitters (K=1024,J=1).

We place four emitters in the RoI. Emitters are placed at [0,0,0],[50,0,0],[0,50,0],[50,50,0] and [25,25,0] (km). We layout four transponders and four receiving arrays as in Figure 3a. The RMSE of *D* emitters, where D=1,2,3,4,5, is obtained from the average of the RMSE of all combinations of *D* emitters in five positions. The RMSEs and CRLBs of different *D* are displayed in Figure 16. The other parameters are set the same as in Table 7.

Figure 16 only gives the RMSEs and CRLBs when D=1,2,3,4, since the RMSE turns out to be unstable and the CRLB turns out to be +∞ when D=5. This can be explained by the algebra principle that the number of unknown emitters cannot be greater than the number of transponders.

The numerical simulations and CRLB results demonstrate that the performance of MP-ML is influenced by the number of emitters. If D≪L, the number of emitters has only a slight effect on the performance of MP-ML, and if D=L, the performance will decline significantly. If D>L, MP-ML cannot find any emitter at all.

## 6. Conclusions

A novel geolocation architecture, termed “Multiple Transponders and Multiple Receivers for Multiple Emitters Positioning System (MTRE)” is proposed in this paper. A Direct Position Determination for Multi-path Propagation positioning (MP-DPD) model and a MUltiple SIgnal Classification algorithm for Multi-path Propagation positioning (MP-MUSIC) were proposed to position the emitters in an MTRE system. To optimize the cost function of the MP-MUSIC efficiently, we proved that the cost function of the MP-MUSIC was a linear and non-negative constrained quadratic convex programming. A algorithm named Active Set Algorithm (ASA) is designed to solve the quadratic convex programming further. Numerical results show that the MP-MUSIC with ASA locates multiple emitters precisely, but the Signal Subspace Projection MUSIC algorithm (SSP-MUSIC) does not. We compared the time consumptions of the Interior Point Algorithm (IPA) and the ASA, as well. ASA consumes only 1.67% more time than IPA.

In the case of time-sensitive positioning, the number of snapshots is not enough. The maximum likelihood estimation algorithm for Multi-path Propagation positioning (MP-ML) maximizes the likelihood function rather than calculating the covariance matrix of the observations to avoid the requirement of a large number of snapshots. We designed an iterative algorithm and proposed the strategy of choosing an initial solution to accelerate the solving of the programming. Numerical simulation results show that MP-ML can approach the Cramér–Rao Lower Bound (CRLB) relative to MP-MUSIC with the same data length, but MP-ML requires more computation time than the MP-MUSIC method.

Furthermore, we discussed the performances of MP-ML with different numbers of receiving arrays and emitters. SPG mentioned in [28] is viewed as a degenerate version of MP-ML (where D=1 and N=1). The numerical results shows that it is worthwhile to increase the number of receiving stations in the sense of a weak signal, although MP-ML increases the hardware costs, communication overhead and computational complexity.

We compared the performances and time consumptions of MP-MUSIC and MP-ML by numerical simulations. MP-ML obtains a more precise position estimation than MP-MUSIC, and MP-MUSIC consumes less time than MP-ML. In a specific positioning application, we choose the appropriate method according to the number of snapshots, the precision requirement and the calculation ability.

MP-ML has the ability of positioning multiple emitters synchronously. If the number of emitters is far less than the number of transponders, the number of emitters has only a slight influence on the positioning performance. If the number of emitters is equal to the number of transponders, the performance will decline significantly. If the number of emitters is more than the number of transponders, MP-ML cannot find any emitter at all.

An MTRE system requires more receiving arrays, more transponders and more computing resources compared to a Single Geolocation Platform (SGP) or a Direction Finding System (DFS). However, an MTRE system can locate multiple emitters synchronously and provides a higher positioning accuracy than SGP and DFS. It is suitable for some cost-insensitive applications, such as military and national security applications.

## Figures and Tables

**Table 1:** Parameter setting in the numerical simulations.

Description	Layout	*R* (m)	λ (m)/*f* (MHz)	*M*	*B* (kHz)	*K*	*J*	SNR (dB)
Baseband signal positioning	A	30	*1157.5/0.26*	11	8	64	100	10
Transponders are close	*B*	30	30/10	11	8	64	100	10
Single antenna of each receiver	A	30	30/10	*1*	8	64	100	10
Standard scenario	A	30	30/10	11	8	64	100	10

**Table 2:** Parameter setting in MP-MUSIC simulations.

Description	Layout	*R* (m)	λ (m)/*f* (MHz)	*M*	*B* (kHz)	*K*	*J*	SNR (dB)
RMSE of MUSIC methods	A	30	1157.5/0.26	11	8	128	100	−15∼15

**Table 3:** Parameter setting in insufficient snapshot scenarios.

Description	Layout	*R* (m)	λ (m)/*f* (MHz)	*M*	*B* (kHz)	*K*	*J*	SNR (dB)
*MP-MUSIC*	A	30	1157.5/0.26	11	8	16	1	−15∼15
*MP-ML*	A	30	1157.5/0.26	11	8	16	1	−15∼15
*CRLB*	A	30	1157.5/0.26	11	8	16	1	−15∼15

**Table 4:** Parameter setting in MP-MUSIC with different J,K combinations.

Description	Layout	*R* (m)	λ (m)/*f* (MHz)	*M*	*B* (kHz)	*K*	*J*	SNR (dB)
MP-MUSIC J,K Combination I	A	30	1157.5/0.26	11	8	*32*	*4*	0∼30
MP-MUSIC J,K Combination II	A	30	1157.5/0.26	11	8	*16*	*8*	0∼30
MP-MUSIC J,K Combination III	A	30	1157.5/0.26	11	8	*8*	*16*	0∼30
MP-MUSIC J,K Combination IV	A	30	1157.5/0.26	11	8	*4*	*32*	0∼30
MP-ML	A	30	1157.5/0.26	11	8	*128*	*1*	0∼30
CRLB	A	30	1157.5/0.26	11	8	*128*	*1*	0∼30

**Table 5:** Parameter setting in simulations of different numbers of snapshots.

Description	Layout	*R* (m)	λ (m)/*f* (MHz)	*M*	*B* (kHz)	K·J	SNR (dB)
*MP-MUSIC*	A	30	1157.5/0.26	11	8	$2^{i}$, (i=4,5,⋯,13)	10
*MP-ML*	A	30	1157.5/0.26	11	8	$2^{i}$, (i=4,5,⋯,13)	10
*CRLB*	A	30	1157.5/0.26	11	8	$2^{i}$, (i=4,5,⋯,13)	10

**Table 6:** Parameter setting in simulations of time consumption.

Description	Layout	*d*	*R* (m)	λ (m)/*f* (MHz)	*M*	*B* (kHz)	SNR	*K*	*J*
MP-ML (KJ=16)	A	1∼4	30	1157.5/0.26	11	8	15	*16*	*1*
MP-MUSIC (KJ=16)	A	1∼4	30	1157.5/0.26	11	8	15	*16*	*1*
MP-ML (KJ=128)	A	1∼4	30	1157.5/0.26	11	8	15	*128*	*1*
MP-MUSIC (KJ=128)	A	1∼4	30	1157.5/0.26	11	8	15	*16*	*8*

**Table 7:** Parameter setting in simulations of different numbers of receivers. SGP, Single Platform Geolocation .

Description	Number of Receivers	*R* (m)	λ (m)/*f* (MHz)	*M*	*B* (kHz)	*K*	SNR (dB)
MP-ML	*2*∼*4*	30	1157.5/0.26	11	8	128	10
CRLB	*1*∼*4*	30	1157.5/0.26	11	8	128	10
SGP	*1*	30	1157.5/0.26	11	8	128	10

**Figure A1:**
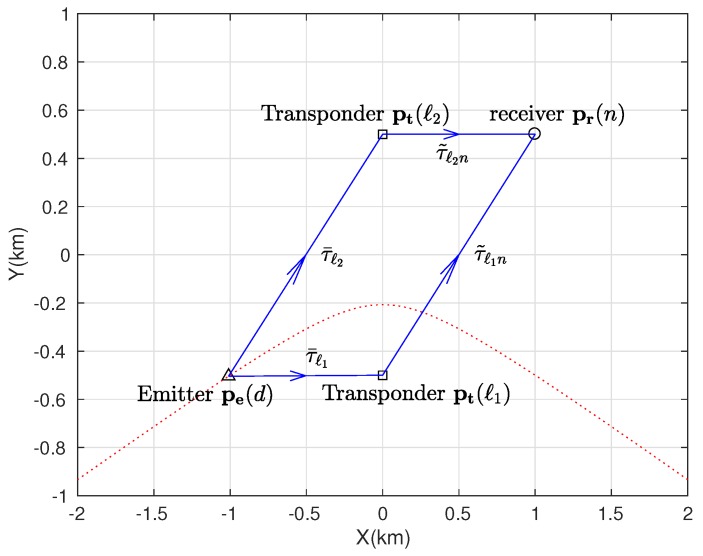
Two paths with the same delay.

## Appendix A. Conditions of a Near Singular Manifold Matrix

We discuss the conditions that make the manifold matrix singular in this section. Define the term of Singular Manifold Candidate (SMC) and Near Singular Manifold Candidate (NSMC) firstly.

**Definition** **A1.** Singular Manifold Candidate (SMC): In a multi-path positioning application, a candidate position $p_{e}$ is named a singular manifold candidate if it satisfies $e^{-j\omega_{k}\left[{\tilde{\tau }}_{\ell_{1}n}+{\bar{\tau }}_{\ell_{1}}\left(p_{e}\right)\right]}=e^{-j\omega_{k}\left[{\tilde{\tau }}_{\ell_{2}n}+{\bar{\tau }}_{\ell_{2}}\left(p_{e}\right)\right]}$, k=1,2,⋯,K, where ${\tilde{\tau }}_{\ell_{i}n},i=1,2$, is the propagation delays from the $\ell_{i}$-th transponder to the n-th receiving array and ${\bar{\tau }}_{\ell_{i}},i=1,2$, is the propagation delay from the candidate position to the $\ell_{i}$-th transponder.

**Definition** **A2.** Center Singular Manifold Candidate (CSMC): In a multi-path positioning application, a candidate position $p_{e}$ is named a singular manifold candidate, if it satisfies $e^{-j\omega_{k}\left[{\tilde{\tau }}_{\ell_{1}n}+{\bar{\tau }}_{\ell_{1}}\left(p_{e}\right)\right]}=e^{-j\omega_{k}\left[{\tilde{\tau }}_{\ell_{2}n}+{\bar{\tau }}_{\ell_{2}}\left(p_{e}\right)\right]}$, k=1,2,⋯,K, where ${\tilde{\tau }}_{\ell_{i}n},i=1,2$, is the propagation delay from the $\ell_{i}$-th transponder to the center of the n-th receiving array and ${\bar{\tau }}_{\ell_{i}},i=1,2$, is the propagation delay from the candidate position to the $\ell_{i}$-th transponder.

The difference of an SMC and a CSMC is that all the antennas satisfy the equation for an SMC, but only the centers of the array satisfy the equation for a CSMC.

**Theorem** **A1.** *If $p_{e}$ is a CSMC, there are at least two paths from $p_{e}$ to a receiving array, which satisfy:*

(A1) $${\tilde{D}}_{\ell_{1,2}n}\left(p_{e}\right)=z\lambda ,z=0,1,2,\cdots ,$$

*where n is the receiving array index, $\ell_{1}$ and $\ell_{2}$ are the indexes of two transponders in two paths, ${\tilde{D}}_{\ell_{1,2}n}≜∥p_{e}-p_{t}\left(\ell_{1}\right){∥}_{F}+∥p_{r}\left(n\right)-p_{t}\left(\ell_{1}\right){∥}_{F}-∥p_{e}-p_{t}\left(\ell_{2}\right){∥}_{F}-{∥p_{r}\left(n\right)-p_{t}\left(\ell_{2}\right)∥}_{F}$ is the difference between the two path lengths, $p_{r}\left(n\right)$ is the center of the n-th receiving array, z is an integer, λ is the Least Common Multiple (LCM) of $\left\{\lambda_{1},\lambda_{2},\cdots ,\lambda_{K}\right\}$ and $\lambda_{k}$ is the wave length of frequency $\omega_{k}$.*

**Proof.** Move the right item of the equation in the CSMC condition to the left:

(A2) $$\begin{array}{c} e^{-j\omega_{k}\left\{\left[{\tilde{\tau }}_{\ell_{1}n}+{\bar{\tau }}_{\ell_{1}}\left(p_{e}\right)\right]-\left[{\tilde{\tau }}_{\ell_{2}n}+{\bar{\tau }}_{\ell_{2}}\left(p_{e}\right)\right]\right\}}=1,\\ \Rightarrow e^{-j\frac{2\pi c}{\lambda_{k}}\left\{\left[{\tilde{\tau }}_{\ell_{1}n}+{\bar{\tau }}_{\ell_{1}}\left(p_{e}\right)\right]-\left[{\tilde{\tau }}_{\ell_{2}n}+{\bar{\tau }}_{\ell_{2}}\left(p_{e}\right)\right]\right\}}=1,\\ \Rightarrow \frac{2\pi c}{\lambda_{k}}\left\{\left[{\tilde{\tau }}_{\ell_{1}n}+{\bar{\tau }}_{\ell_{1}}\left(p_{e}\right)\right]-\left[{\tilde{\tau }}_{\ell_{2}n}+{\bar{\tau }}_{\ell_{2}}\left(p_{e}\right)\right]\right\}\;\mathrm{mod}\;2\pi =0,\\ \Rightarrow \frac{{\tilde{D}}_{\ell_{1,2}n}\left(p_{e}\right)}{\lambda_{k}}\in Z,k=1,2,\cdots ,K,\\ \Rightarrow {\tilde{D}}_{\ell_{1,2}n}\left(p_{e}\right)=z\lambda ,z=0,1,2,\cdots , \end{array}$$

where $\lambda_{k}=\frac{2\pi c}{\omega_{k}}$ is the wave length of frequency $\omega_{k}$, $c=3.0\times 10^{8}$ m/s is the light speed constant, ${\tilde{D}}_{\ell_{1,2}n}≜∥p_{e}-p_{t}\left(\ell_{1}\right){∥}_{F}+∥p_{r}\left(n\right)-p_{t}\left(\ell_{1}\right){∥}_{F}-∥p_{e}-p_{t}\left(\ell_{2}\right){∥}_{F}-{∥p_{r}\left(n\right)-p_{t}\left(\ell_{2}\right)∥}_{F}$ is the difference between the two path lengths, $p_{r}\left(n\right)$ is the center of the *n* th receiver and Z is the integer set. Denote λ as the Least Common Multiple (LCM) of $\left\{\lambda_{1},\lambda_{2},\cdots ,\lambda_{K}\right\}$. ☐

In particular, we set z=0 and obtain ${\tilde{D}}_{d\ell_{1,2}n}=0$ (see Figure A1) (in fact, $p_{e}\left(d\right)$ on the hyperbolic satisfies ${\tilde{\tau }}_{\ell_{1}n}+{\bar{\tau }}_{\ell_{1}}={\tilde{\tau }}_{\ell_{2}n}+{\bar{\tau }}_{\ell_{2}}$).

**Theorem** **A2.** If $p_{e}$ is a CSMC and $a_{\ell_{1}n}\left(k\right)\approx a_{\ell_{2}n}\left(k\right),k=1,2,\ldots ,K$, the candidate emitter position $p_{e}$ makes the manifold matrix near singular.

**Proof.** In a multi-path positioning model, Γ(k) in (16) is defined as:

(A3) $$\Gamma \left(k\right)≜\tilde{A}\left(k\right)V\left(k\right),$$

where Γ(k) is a block diagonal matrix:

(A4) $$\Gamma \left(k\right)={\left[\begin{array}{cccc} \Gamma_{1}\left(k\right) & 0 & \cdots & 0\\ 0 & \Gamma_{2}\left(k\right) & \cdots & 0\\ \vdots & \vdots & \ddots & \vdots \\ 0 & 0 & \cdots & \Gamma_{N}\left(k\right) \end{array}\right]}_{MN\times LN}$$

The *n*-th block $\Gamma_{n}\left(k\right)$ in the diagonal is a matrix with a size of M×L. The *ℓ*-th column of $\Gamma_{n}\left(k\right)$ is defined as $e^{-i\omega_{k}\left({\tilde{\tau }}_{\ell n}+{\bar{\tau }}_{\ell }\right)}$, where $\tau_{\ell n}$ is an M×1 column vector representing the propagation delays from the *ℓ*-th transponder to the *M* antennas in the *n*-th receiving station. Notice that:

(A5) $$e^{-i\omega_{k}\left({\tilde{\tau }}_{\ell n}+{\bar{\tau }}_{\ell }\right)}=e^{-i\omega_{k}\left({\tilde{\tau }}_{\ell n}-\tau_{\ell n}+\tau_{\ell n}+{\bar{\tau }}_{\ell }\right)}=e^{-i\omega_{k}\left({\tilde{\tau }}_{\ell n}-\tau_{\ell n}\right)}e^{-i\omega_{k}\left(\tau_{\ell n}+{\bar{\tau }}_{\ell }\right)}≜a_{\ell n}\left(k\right)e^{-i\omega_{k}\left(\tau_{\ell n}+{\bar{\tau }}_{\ell }\right)}$$

where $\tau_{\ell n}$ is the propagation delay from the *ℓ*-th transponder to the center of the *n*-th receiving array and $a_{\ell n}\left(k\right)=e^{-i\omega_{k}\left({\tilde{\tau }}_{\ell n}-\tau_{\ell n}\right)}$ represents the array response of the *n*-th receiving station from the *ℓ*-th transponder. If $p_{e}$ is a CSMC that satisfies ${\tilde{D}}_{\ell_{1,2}n}=z\lambda$ and $a_{\ell_{1}n}\left(k\right)\approx a_{\ell_{2}n}\left(k\right)$, we get that $e^{-i\omega_{k}\left({\tilde{\tau }}_{\ell_{1}n}+{\bar{\tau }}_{\ell_{1}}\right)}\approx e^{-i\omega_{k}\left({\tilde{\tau }}_{\ell_{2}n}+{\bar{\tau }}_{\ell_{2}}\right)}$ from (A5). In this case, the $\ell_{1}$-th column of matrix $\Gamma_{n}\left(k\right)$ is approximately equal to the $\ell_{2}$-th column. Because there are two columns in the matrix Γ(k) that are almost equal, Γ(k) is a near singular matrix. ☐

## Appendix B. Convergence of the Iterative Algorithm

The proof of Theorem 2:

**Proof.** Denote a specific value of α by $\alpha_{i}$. $\bar{f}\left(k\right)$ and $\bar{C}\left(k\right)$ are vectors with real values. The following inequality is always true,

(A6) $$∥\bar{f}{\left(k\right)}^{T}\alpha {\left[\alpha^{T}\bar{C}\left(k\right)\alpha \right]}^{-\frac{1}{2}}-\bar{f}{\left(k\right)}^{T}\alpha_{i}{\left[\alpha_{i}^{T}\bar{C}\left(k\right)\alpha_{i}\right]}^{-1}{\left[\alpha^{T}\bar{C}\left(k\right)\alpha \right]}^{\frac{1}{2}}{∥}_{F}^{2}\geq 0.$$

After expanding the expression in (A6) and re-arranging the terms,

(A7) $$\begin{array}{cc} \bar{f}{\left(k\right)}^{T}\alpha {\left[\alpha^{T}\bar{C}\left(k\right)\alpha \right]}^{-1}\alpha^{T}\bar{f}\left(k\right)\geq & 2\{\bar{f}{\left(k\right)}^{T}\alpha {\left[\alpha_{i}^{T}\bar{C}\left(k\right)\alpha_{i}\right]}^{-T}\alpha_{i}^{T}\bar{f}\left(k\right)\}\\ & -\bar{f}{\left(k\right)}^{T}\alpha_{i}{\left[\alpha_{i}^{T}\bar{C}\left(k\right)\alpha_{i}\right]}^{-1}\left[\alpha^{T}\bar{C}\left(k\right)\alpha \right){\left(\alpha_{i}^{T}\bar{C}\left(k\right)\alpha_{i}\right]}^{-T}\alpha_{i}^{T}\bar{f}\left(k\right). \end{array}$$

Defining $w\left(k\right)≜\bar{f}{\left(k\right)}^{T}\alpha_{i}{\left[\alpha_{i}^{T}\bar{C}\left(k\right)\alpha_{i}\right]}^{-1}$, and summing (A7) over *k*,

(A8) $$\begin{array}{cc} Q(\eta ) & =H(\alpha )\\ & =-\sum_{k=1}^{K}\bar{f}{\left(k\right)}^{T}\alpha {\left[\alpha^{T}\bar{C}\left(k\right)\alpha \right]}^{-1}\alpha^{T}\bar{f}\left(k\right)\\ & \leq -2\sum_{k=1}^{K}\left\{{\bar{f}}^{T}\left(k\right)\alpha w^{T}\left(k\right)\right\}+\sum_{k=1}^{K}w\left(k\right)\left[\alpha^{T}\bar{C}\left(k\right)\alpha \right]w^{T}\left(k\right). \end{array}$$

Notice that:

(A9) $$w\left(k\right)\alpha^{T}={\bar{\alpha }}^{T}W\left(k\right),$$

where $\bar{\alpha }$ is defined in (38), and it is viewed as a reshaped form of the α. α is defined in (5), and:

(A10) $$W\left(k\right)≜I_{N}⊗\mathrm{diag}\left\{w\left(k\right)\right\}⊗I_{L},$$

where $I_{L}$ is an identify matrix with a size of L×L and $I_{N}$ is an identify matrix with a size of N×N. Substitute (A9) into (A8):

(A11) $$\begin{array}{cc} H(\bar{\alpha }) & \leq -2\left[\sum_{k=1}^{K}{\bar{f}}^{T}\left(k\right)W^{T}\left(k\right)\right]\bar{\alpha }+{\bar{\alpha }}^{T}\left[\sum_{k=1}^{K}W\left(k\right)\bar{C}\left(k\right)W^{T}\left(k\right)\right]\bar{\alpha }. \end{array}$$

The eigenvalue decomposition of the second item of the right part is denoted by:

(A12) $$\begin{array}{cc} \sum_{k=1}^{K}W\left(k\right)\bar{C}\left(k\right)W^{T}\left(k\right) & ={\bar{U}}^{T}\Sigma \bar{U}\\ & =U^{T}U, \end{array}$$

where $U=\bar{U}\Sigma^{\frac{1}{2}}$. The right part of (A11) is simplified by:

(A13) $$\begin{array}{c} -2\left[\sum_{k=1}^{K}\bar{f}{\left(k\right)}^{T}W{\left(k\right)}^{T}\right]\bar{\alpha }+{\bar{\alpha }}^{T}\left[\sum_{k=1}^{K}W\left(k\right)\bar{C}\left(k\right)W{\left(k\right)}^{T}\right]\bar{\alpha }\\ =||FU^{-1}-{\bar{\alpha }}^{T}U^{T}{\left|\right|}^{2}-{\mathrm{FU}}^{-1}U^{-T}F^{T}\\ ≜||Y-{\bar{\alpha }}^{T}X{\left|\right|}^{2}-Z, \end{array}$$

where:

$$\begin{array}{cc} F & ≜\sum_{k=1}^{K}\bar{f}{\left(k\right)}^{T}W{\left(k\right)}^{T},\\ Y & ≜{\mathrm{FU}}^{-1},\\ X & ≜U^{T},\\ Z & ≜{\mathrm{YY}}^{T}. \end{array}$$

Denote:

(A14) $$\begin{array}{c} G\left(\alpha_{i},\bar{\alpha },P^{e}\right)≜||Y-{\bar{\alpha }}^{H}X{\left|\right|}^{2}-Z. \end{array}$$

For a given $\alpha_{i}$, $G(\alpha_{i},\bar{\alpha },p^{e})$ is the upper bound of the cost function Q(η). The cost function defined in (A14) is viewed as a relaxed programming of the original programming:

(A15) $${\bar{\alpha }}_{i+1}=\arg\min_{\bar{\alpha }}G\left(\alpha_{i},\bar{\alpha },p_{e}\right)=||Y-{\bar{\alpha }}^{H}X{\left|\right|}^{2}-Z,$$

$$\begin{array}{c} s.t.\;\bar{\alpha }\geq 0. \end{array}$$

Since ${\bar{\alpha }}_{i+1}$ is the optimal solution of the relaxed programming,

(A16) $$Q\left(\eta_{i+1}\right)=H\left({\bar{\alpha }}_{i+1}\right)\leq G\left(\alpha_{i},{\bar{\alpha }}_{i+1},p_{e}\right)\leq G\left(\alpha_{i},{\bar{\alpha }}_{i},p_{e}\right)=H\left({\bar{\alpha }}_{i}\right)=Q\left(\eta_{i}\right),$$

☐
